# A Meta-Analysis of Global Urban Land Expansion

**DOI:** 10.1371/journal.pone.0023777

**Published:** 2011-08-18

**Authors:** Karen C. Seto, Michail Fragkias, Burak Güneralp, Michael K. Reilly

**Affiliations:** 1 Yale School of Forestry and Environmental Studies, Yale University, New Haven, Connecticut, United States of America; 2 International Human Dimensions Programme on Global Environmental Change, Urbanization and Global Environmental Change Project, Global Institute for Sustainability, Arizona State University, Tempe, Arizona, United States of America; 3 Department of Environmental Earth System Science, Stanford University, Stanford, California, United States of America; Universidade de Vigo, Spain

## Abstract

The conversion of Earth's land surface to urban uses is one of the most irreversible human impacts on the global biosphere. It drives the loss of farmland, affects local climate, fragments habitats, and threatens biodiversity. Here we present a meta-analysis of 326 studies that have used remotely sensed images to map urban land conversion. We report a worldwide observed increase in urban land area of 58,000 km^2^ from 1970 to 2000. India, China, and Africa have experienced the highest rates of urban land expansion, and the largest change in total urban extent has occurred in North America. Across all regions and for all three decades, urban land expansion rates are higher than or equal to urban population growth rates, suggesting that urban growth is becoming more expansive than compact. Annual growth in GDP per capita drives approximately half of the observed urban land expansion in China but only moderately affects urban expansion in India and Africa, where urban land expansion is driven more by urban population growth. In high income countries, rates of urban land expansion are slower and increasingly related to GDP growth. However, in North America, population growth contributes more to urban expansion than it does in Europe. Much of the observed variation in urban expansion was not captured by either population, GDP, or other variables in the model. This suggests that contemporary urban expansion is related to a variety of factors difficult to observe comprehensively at the global level, including international capital flows, the informal economy, land use policy, and generalized transport costs. Using the results from the global model, we develop forecasts for new urban land cover using SRES Scenarios. Our results show that by 2030, global urban land cover will increase between 430,000 km^2^ and 12,568,000 km^2^, with an estimate of 1,527,000 km^2^ more likely.

## Introduction

Earth's land surface is a finite resource that is central to human welfare and the functioning of the Earth system. Globally, human activities are transforming the terrestrial environment at unparalleled rates and scales. Croplands and pastures now cover approximately 40% of the land surface, nearly equal in area to that covered by forests [1]. On the continuum of anthropogenic activities, urbanization is the most irreversible and human-dominated form of land use. Urbanization results in changes in land-cover, hydrological systems, biogeochemistry, climate, and biodiversity [2]. Worldwide, urban expansion is one of the primary drivers of habitat loss, and species extinction [3]. In many developing countries, urban expansion is taking place on prime agricultural land [4]. In the United States, urban expansion in the form of housing development is a major threat to protected areas [5]. Urban areas affect their local climate through the modification of surface albedo and evapotranspiration, and increased aerosols and anthropogenic heat sources, resulting in elevated temperatures [6] and changes in precipitation patterns [7], [8]. The spatial form of cities, especially urban transportation infrastructure and residential density, affects travel demand [9], energy consumption [10], and automobile use [11].

At the same time, urbanization presents opportunities for efficient resource use and mitigating climate change. Compact urban development coupled with high residential and employment densities can reduce energy consumption, vehicle miles traveled, and carbon dioxide emissions [12]. Increasing urban albedo could offset greenhouse gas emissions [13]. Furthermore, per capita greenhouse emissions of urban areas are often lower than national averages [14].

Despite the importance of urban land use to local and global environmental change, the rate and magnitude of urban expansion have not been quantified at global scales. Our understanding of urban change at global scales is primarily based on United Nations population figures, but these statistics do not provide information on the distribution, pattern, and scale of urban land use change. Satellite-based efforts at mapping global urban extents fail to agree on the size and pattern of urban land use, with estimates ranging from 0.2% to 2.4% of terrestrial land surface circa 2000 [15]. Importantly, these global-scale efforts do not track the growth of urban extent. Here, we present a meta-analysis of studies that examine the rate and magnitude of urban land expansion worldwide.

## Methods

We reviewed the English language literature for studies that monitor urban land-use change using satellite or airborne remotely sensed data published between 1988 and December 2008. We searched the ISI Web of Science database using keywords that focused on satellite or remote sensing data, urbanization, cities, the built environment, and land-cover and land-use change (see Text S1 for full search strings and Table S1 for a list of journals). There is no uniform definition of urban worldwide, and most countries define urban according to criteria pertaining to some aspect of a region's population, economy, or built infrastructure. Due to this variation in definition, it is difficult to compare urban areas across countries using demographic datasets. For this reason, remote sensing-based studies offer an advantage because the definition of urban by satellite studies is more uniform across regions. In this study, urban is defined as land cover and land use, impervious surfaces, and other manifestation of the built environment; it does not measure population or population density. In order to be included in our analysis, the study had to meet the following four criteria:

1. Study must quantify the urban area extent for at least in one point in time.
2. Study must quantify either the rate or amount of urban land expansion over a specific period of time.
3. Study area extent must be at city, metro, or regional scale (<100,000 km^2^).
4. Study must not repeat the results presented in another paper.

**Figure 1:**
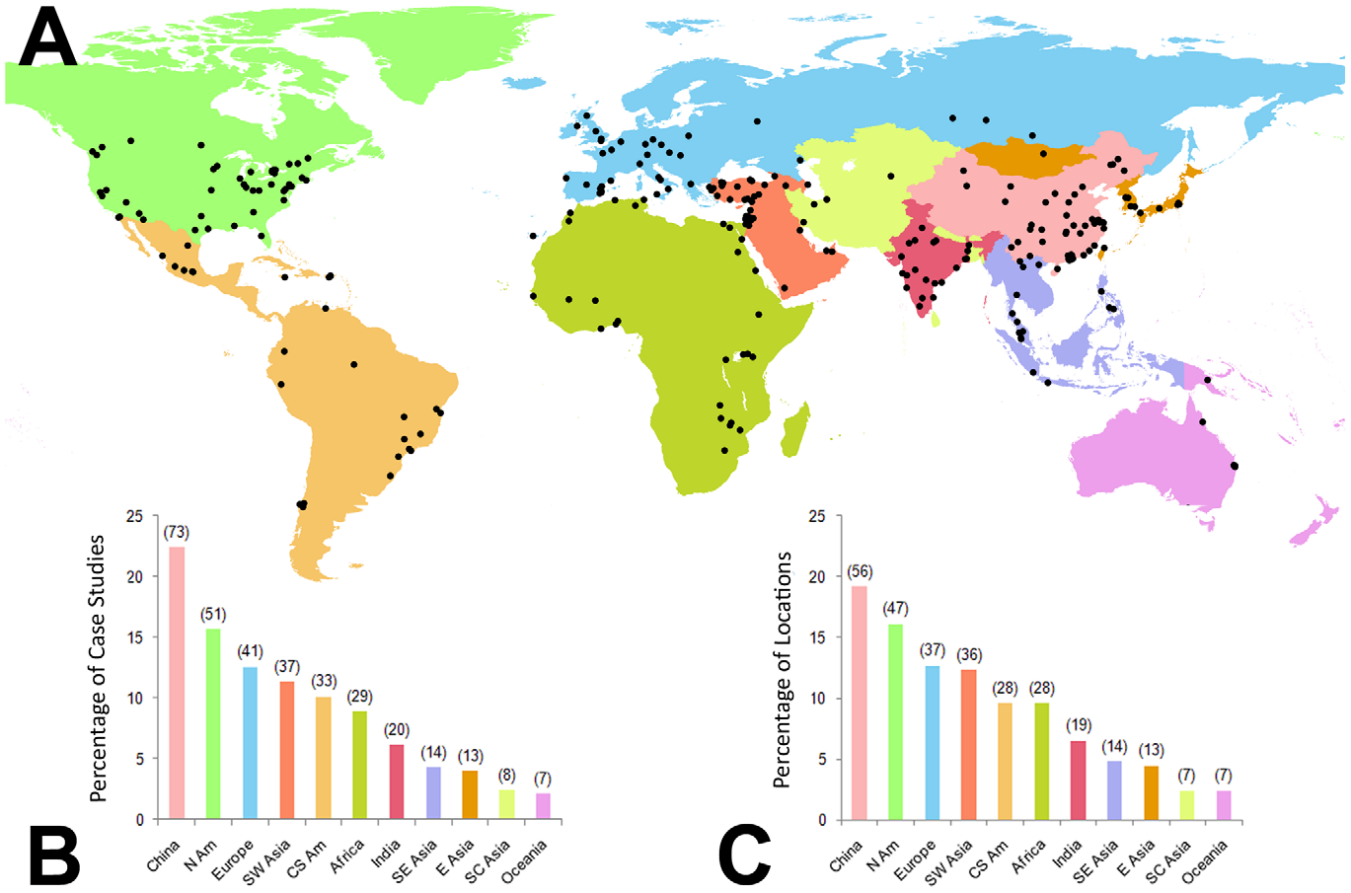
Geographical distribution of case studies and their locations. **A**, Locations of case studies. **B**, Studies by region. Numbers in parentheses are the number of case studies for each region. The total number of case studies is 326. **C**, Locations by region. Numbers in parentheses are the number of locations for each region. The total number of unique locations is 292. There are more case studies than geographic locations because there may be multiple case studies on a single location. The color-coding for the map corresponds to the bar charts.

The literature review generated more than 1,000 papers. Among these, we filtered those that met criteria 1 and 2, which resulted in 264 papers. We further narrowed this set of papers to those that meet criteria 3 and 4, which yielded 180 papers. In addition to this set of peer-reviewed papers, we reviewed and included a World Bank study that was similar in method and scientific rigor. All of the papers included in the meta-analysis are listed in Text S2. There are more case studies than research papers because some papers include several case studies. There are more case studies than geographic locations because there may be multiple case studies on a single location. The regional breakdown follows the United Nations (UN) defined world macro regions except Asia, which was further geographically disaggregated into UN regions, and China and India were treated as individual regions. The 181 papers include 326 case studies of 292 unique geographic locations distributed across 67 countries in all continents except Antarctica (Figures S1, S2, S3 and S4). 19% and 16% of the study locations are in China and North America, respectively (Figure 1). The case studies capture only a portion of the world's largest urban agglomerations circa 2007; only 48 of the world's 100 currently largest urban areas have been studied with findings in peer reviewed journals.

We synthesized the studies and calculated four measures of urban expansion for eleven geographic regions: 1) total area extent of urban expansion between 1970 and 2000; 2) total area extent of urban expansion by decade between 1970 and 2000; 3) average annual percentage rates of urban expansion between 1970 and 2000; and 4) average annual percentage rates of urban expansion by decade between 1970 and 2000. We extracted and analyzed measures of urban land expansion from the individual case studies. The measures were converted into a standard metric, the annual rate of urban land expansion, calculated as AGR  =  100*((UE_end_/UE_start_)^(1/d)^-1) where *UE_start_* is the extent of the urban area at the initial time period, *UE_end_* the extent of the study at the final time period and *d* the time span of the study in years. In our calculations of this effect size, we accounted for differences in the size of time intervals between the monitoring of urban areas. As a simplifying assumption and to be able to include those papers that only report the year an image is taken, we did not use the exact date but the first day of the year an image was acquired in our calculations. We recognize that the image processing algorithms used to identify urban will vary among studies. However, the extraction of rates of change from two or more time points classified by the same study mitigates the variation in classification methodologies.

The size of study areas, when not reported explicitly in the text, was gathered from tables, figures and maps. For the decadal estimates, the starting year of each decade was used to estimate total urban land area and the change in urban land area that occurred in each decade. Following our aggregation method for the regions, we calculated the aggregate average annual rate of change for each region for both the decadal periods and the entire study period, 1970–2000. We used nonparametric bootstrapping methods to estimate a measure of uncertainty over the rates of change and report the quartiles of the bootstrap distribution of the means of each region and time period (see Text S3 for details of the meta-analysis methodology).

For the protected area (PA) analysis, we used the 2009 World Protected Area Database and included only the terrestrial protected areas with International Union of Conservation of Nature (IUCN) status (http://www.wdpa.org). We calculated the distance from each PA to urban areas in the meta-analysis. We used geographic coordinate information provided in each case study, and when this information was not available, we determined the approximate coordinates of the central urban area from the satellite images in the study and Google Earth. As an approximation, we buffered the urban coordinates to create circular regions equal to the largest extent as reported in the respective studies of each urban area. Next, we created buffer zones of 10 km around all terrestrial protected areas. We then calculated the urban areas that fall –wholly or in part—within the buffer zones around the protected areas and those that do not. This created two groups of urban locations in our meta-analysis. Finally, we calculated the average rates of change of the two groups. We repeated the analyses with urban buffer zones of 5km and 15 km and found our results to be robust to the value of the buffer distance.

In order to evaluate whether urban expansion was more likely in coastal areas, we used the low-elevation coastal zone (LECZ) map created by Socio-Economic Data and Applications Center (SEDAC) at the Center for International Earth Science Information Network (CIESIN). Similar to the PA analysis, we identified those urban locations that fall into the LECZ wholly or in part and those that do not. Then we calculated the average rates of change of the two groups. We used one-tailed tests in the LECZ and PA analyses based on empirical evidence and theoretical work that suggests that coastal settlements present economies of agglomeration through their geographic advantage and thus experience greater economic activity and larger expanses of physical development. Similarly, we expect less economic activity and urban growth close to internationally designated protected areas.

We used a multivariate regression on the pooled dataset to model the global rate of urban land expansion. We used the decadal estimate of urban land expansion for each city, and dropped 14 cases where there was a negative urban expansion rate or which were largely rural locations, resulting in 360 observations. We selected a range of independent variables based on urban theory and models, representing the major forces that drive the physical expansion of urban land cover.

Our dependent variable was a single annual rate for each decadal period in each study. The independent variables were developed through the following methods. The *population growth rate* (% annual) was developed primarily by taking decadal populations totals from J. Vernon Henderson's World Cities database (http://econ.pstc.brown.edu/faculty/henderson/worldcities.html) and converting them to annualized percentage growth rates for each decade in each city. For a number of smaller cities that are not included in that database, similar growth rates were developed using the UN World Urbanization Prospects (2008) dataset. *Gross Domestic Product* (GDP) was collected at the national level from the Penn World Table v6.2 database. A single annualized GDP per capita percentage growth rate was calculated by country for each decade. Because of the country's vast size and economic variation, GDP was collected at the provincial level for China from Chinese Yearbooks [16]. A variety of country groupings (based on wealth and policy) and variable transforms (e.g. growth squared) were also tested.

The best predictive power for GDP's influence was established by dividing countries into low, middle, and high income categories with a separate high income category for three countries with national policies (e.g. taxation and subsidies) favoring automobile use, the United States, Canada, and Australia. A squared term offered higher predictive power for these countries and the middle income countries while an unmodified GDP variable offered the best fit for the grouping of all other high income countries. In no models tested did the low income GDP growth rate significantly impact the urban expansion rate. Overall, higher population and economic growth rates are expected to lead to higher rates of urban expansion; the former through the need for land for residential use and supporting activities, the latter through both the need for economically productive land and the tendency for wealthier households to consume more land and to purchase more goods and services. This final pathway suggests interactive effects; a number of interaction terms between explanatory variables were tested but none were found to be statistically significant. Additionally, the influence of GDP growth on urban expansion is expected to be higher for middle income countries than for wealthier countries because the bulk of economic growth in these countries is driven by the manufacturing sector which has large land requirements, as opposed to the service sector growth dominating the high income developed world. Negative values of both population and economic growth rates were capped at 0.

In addition to the fundamental population and economic drivers, we also tested other policy, space, and time factors. An indicator variable called *Farm Subsidy* was collected from the UN Food and Agriculture Organization (FAO) and a *Coastal Zone Location* was used for cities in the low-elevation coastal zone. Trends over time were assessed by comparing the average rate of expansion by decade as well as the rate for a variety of temporal subsets. The only significant trend was that the 1980s experienced more land expansion than other years, *ceteris paribus*, so observations from that period are marked by the *1980s indicator* variable. Finally, the *Study Area Size* variable was developed with data from the papers. All factors that correlated with a profitable agricultural sector were expected to lessen the rate of urban expansion by making the use of land for agriculture more profitable than urban development for non-urban locations. The overall effects of a low-elevation coastal zone location and temporal changes in expansion rates were both uncertain. Finally, larger study areas were expected to have lower rates of urban expansion because urban growth is highly localized in nature and we expected that the authors of studies over smaller areas would be more likely to choose rapid growth locations. Other variables examined without finding statistical significance were: status as a national-level center of government activity, annual temperature extremes, agricultural productivity, the extent of agricultural irrigation in the region, and topography.

Following the development of the model, we forecasted future urban expansion using rates of population and GDP drawn from the downscaled projections developed at Center for International Earth Science Information Network (CIESIN). Total population growth was translated into urban population growth using regional proportions from the UN World Urbanization Prospects (2008) dataset. These rates were annualized and squared where appropriate. The *Farm Subsidy* and *Coastal Zone Location* variables were given a single value for each region based on the proportion of study areas that are located in countries with farm subsidies in year 2000 and low-elevation coastal zones, respectively. The *1980s dummy variable* and the *Study Area Size* variable were not included in the prediction equation.

We forecasted potential global urban land change in the next two decades by conducting a simple exercise. We developed four urban land expansion scenarios based on the Special Report on Emissions and Scenarios (SRES) Scenarios available through CIESIN (http://sres.ciesin.columbia.edu/). The four SRES Scenarios, A1, A2, B1, and B2, were generated at the UN regional level for 2030 based on the global population and GDP projections [17], [18]. The A1 storyline is characterized by high economic growth and low population growth; the A2 storyline is characterized by lower economic development and high population growth; storyline B1 is considered a “sustainable development” scenario with moderate economic growth and low population growth; the B2 storyline has lower economic development than B1 and stabilizing population growth projections. For each of the four scenarios, we created a new dataset to forecast urban land expansion. All variables other than those related to population and GDP remained constant in all four scenarios. We used the coefficients derived in the benchmark model and each of the four population/GDP scenario datasets to predict four sets of Annual Rate of Change (ARC) of urban expansion for each UN region for successive 5-year intervals up to the year 2030. We then applied the four sets of aggregate regional predicted ARC of urban expansion to the three estimates of the 2000/2001 global urban land cover from the Global Rural-Urban Mapping Project (GRUMP) at the Center for International Earth Science Information Network (CIESIN) of the Earth Institute at Columbia University, NASA's Moderate Resolution Imaging Spectroradiometer Urban Land Cover (MODIS), and the European Commission's Global Land Cover 2000 (GLC00). This produced a range of estimates for the global urban land cover in 2030 based on the three different assumptions about the initial urban land cover in 2000/2001. With this preliminary model, a few estimates exceeded the amount of land available in certain regions when using the GRUMP data as the initial urban land cover. Although this is partly due to the possible overestimation of the existing urban land cover in the GRUMP dataset, it is primarily because of the preliminary nature of this exercise, which simply extrapolates a model that considers limited amount of factors based on imperfect data out to 2030 without accounting for other potential factors such as the increasing densification of urban development as land becomes scarce. Although it is a simple exercise, it is the best available forecast of global urban land cover.

## Results

**Figure 2:**
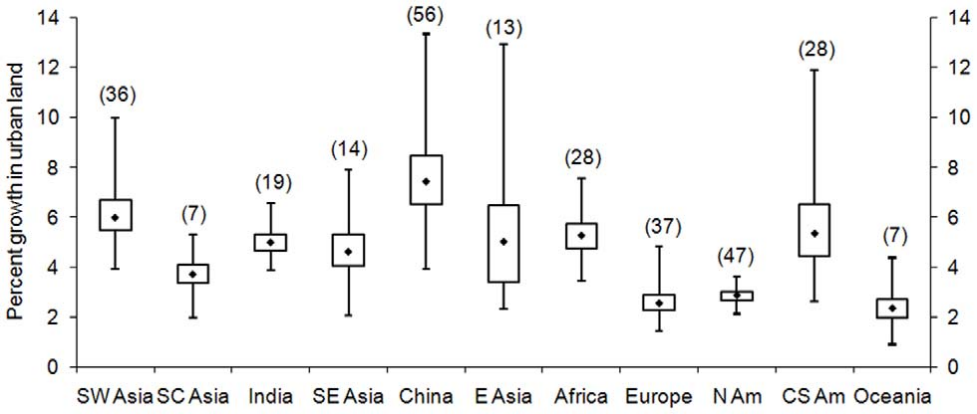
Average annual rates of urban expansion by region (1970–2000). Box plots show the median, 1^st^ and 3^rd^ quartiles, minimum and maximum values of bootstrapped average annual rates of urban expansion by region.

Our results show considerable variation in the rates of urban expansion over the study period, with the highest rates in China followed closely by Southwest Asia (Figure 2). Average rates of urban expansion are lowest for Europe, North America, and Oceania. Variations in urban expansion rates point to differences in national and regional socio-economic environments and political conditions. This is particularly evident in the case of China, where annual rates of urban land expansion vary from 13.3% for coastal areas to 3.9% for the western regions. On the other hand, the range of urban growth rates in North America is more evenly distributed, from 3.9% to 2.2%.

Total change in urban extent for the meta-analysis case studies was 58,000 km^2^ for the period 1970 to 2000. This growth in urban land area is equivalent to 1.3 times the size of the country of Denmark, or approximately 1.56% to 3.89% of the global urban land area in 2000. Reported total urban land conversion was highest in North America, but this could reflect a sampling bias because 16% of the urban areas in the meta-analysis were located in North America. Indeed, the geographic distribution of the meta-analysis case studies indicates that some of the largest cities worldwide are not being studied in terms of their changing urban land extent. In particular, five of the world's largest cities by population, Dhaka, Karachi, Kolkata, Jakarta, and Delhi, were not represented in the meta-analysis case studies.

About 34% (99 out of 292) of the locations in the meta-analysis fall within 10m of low elevation coastal zones (LECZ). For these urban areas, the average rate of urban land expansion from 1970 to 2000 is greater than 5.7%, and statistically higher than urban areas elsewhere (one-tailed p = 0.04228). Given the impacts of climate change and projections of geographically uneven levels in sea level rise and storm surges [19], our results show that humanity has unknowingly been increasing the vulnerability of its urban populations. Almost half of the meta-analysis case studies (47%) are within 10 km of a terrestrial protected area with IUCN status listed in the World Database of Protected Areas. The average annual rate of urban land expansion of these cities from 1970 to 2000 is greater than 4.7% and not statistically different from growth rates of urban areas away from protected areas (one-tailed p = 0.22). Taken together, these results show that urban land expansion is as likely to take place near protected land as elsewhere, and that being near a protected area does not necessarily slow the rate of urban land conversion.

**Figure 3:**
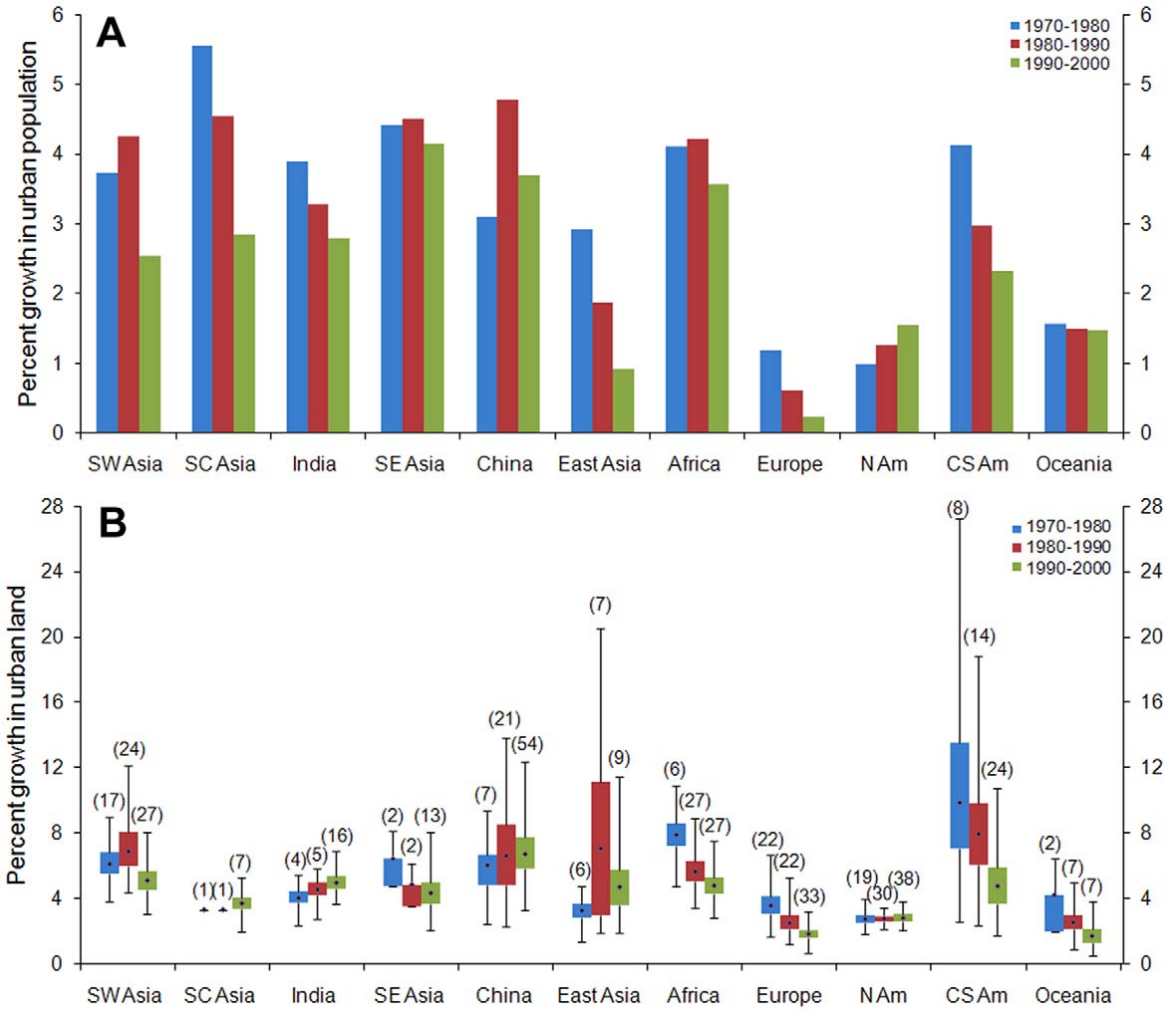
Comparison of two different urban growth measures by region and by decade. Annual rates of **A**, urban population change and **B**, urban land expansion. Population data are aggregated from individual countries to the geographic regions in the meta-analysis. Average annual rate of urban land change is based on the case studies in the meta-analysis. Box plots in **B** show the median, 1^st^ and 3^rd^ quartiles, minimum and maximum values of bootstrapped average annual rates of urban expansion by region.

Across all regions and for all three decades, urban land expansion rates are higher than or equal to urban population growth rates (Figure 3). Nowhere is there evidence of a global increase in urban land use efficiency or urban population density, as defined by the change in urban population per unit change in urban land, suggesting expansive urban growth globally. Rates of urban land expansion by decade reveal three distinct typologies: declining annual rates across the decades (Central and South America, Europe, Oceania, and Africa), no trend (China, North America, and India), and uneven trajectories (Southwest Asia, South East Asia, and East Asia) (Figure 3b). Declining rates of urban land expansion is expected for regions such as South America and Europe, which were already highly urbanized (in terms of percentage of population living in urban areas) in 1970s, with urban population levels of 57% and 63%, respectively. In contrast, declining rates of urban land change are surprising for Africa, where urban population levels were only 24% in 1970. While Africa has consistently higher average rates of urban land expansion than North America, the total urban extent is greater in North America.

Our regression model of global urban expansion shows that every additional percent of annual urban population growth rate increases the annual urban expansion rate by 0.563 percentage points (Table 1). The annual GDP per capita growth rate (squared in middle income countries and high income countries with policies that favor automobile use to show diminishing returns to income) increases the rate of urban land expansion by 0.046 percentage points in China, 0.130 percentage points in middle income countries other than China, 0.980 percentage points in most high income countries, and 0.430 percentage points in countries with policies that favor automobile uses (U.S., Canada, and Australia). These results indicate that high income countries experience more urban land expansion as a function of income than middle income countries. The presence of farm subsidies drives down the annual urban expansion rate by 2.43 percentage points and an urban area located in the coastal zone drives the rate of urban land expansion up by 0.829 percentage points compared to non-coastal zones. During the 1980s, the urban areas reviewed in the meta-study experienced a higher rate of urban expansion—by a 1.347 percentage points—compared with other years. No other temporal effects were significant in the model. Finally, as the study area size increases, the annual urban expansion rate decreases. Although the result is statistically significant, the magnitude of the effect is very small.

**Table 1:** Regression Model Results: Factors Influencing Global Annual Percent Expansion.

*Variable*	*Coef.*	*Std. Err.*
Population growth rate (% annual)	0.563***	0.129
Middle income (China excluded) GDP growth rate squared (% annual)	0.130***	0.0355
China GDP growth rate squared (% annual)	0.046***	0.00614
Automobile-oriented high income GDP growth rate squared (% annual)†	0.430***	0.140
Other high income GDP growth rate (% annual)	0.980**	0.433
Farm subsidy	-2.430***	0.884
Coastal zone location	0.829	0.514
1980s indicator	1.347**	0.559
Study area size	-0.0000479**	0.0000225
Constant	2.273	0.526

Notes:

†the group consists of the U.S., Canada, and Australia;

*indicates significant at α = 0.1;

**indicates significant at α = 0.05;

***indicates significant at α = 0.01.

We used regional averages in the regression model to examine the varying influence of likely factors behind urban land expansion across the regions (Table 2). In China, the average annual population growth rate in the meta-analysis is 2.34% with an average annual GDP per capita growth rate of 9.21%. The average modeled Chinese city has an annual urban land expansion growth rate of 7.48% with approximately 18% of that associated with population increase and just over 50% associated with economic growth. The model results for India shows on average a 4.84% urban land expansion growth rate with 30% from population growth and around 23% from growth in GDP per capita. For Africa, a 4.32% urban expansion rate is 43% attributable to population growth while GDP growth does not demonstrate a significant relationship. In North America, the mean population and economic growth rates are 1.53% and 5.19%, respectively. The rate of urban land expansion for the average North American city was 3.31%, with 28% related to population growth and 72% related to GDP growth. For Europe, the model predicts that the average city will have an annual urban expansion rate of 2.50% with around 86% attributed to GDP growth and 4% attributed to population growth. Globally, the “average” city in the study exhibited an urban population growth rate of 2.18% and urban land expansion growth rate of 4.84%. This indicates that each city in the study added almost 46,000 urban dwellers per year and approximately 13.5 km^2^ of new urban land.

**Table 2:** Percentage of urban land expansion explained by population or GDP growth by region.

*Location*	*Average annual urban expansion growth rate*	*Approximate percent of urban land expansion attributed to*	
		*Population* *growth rate*	*GDP per capita* *growth rate*
China	7.48	18	53
India	4.84	30	23
Africa	4.32	43	Not significant
North America	3.31	28	72
Europe	2.50	4	86

Our forecasts of global urban land cover for 2030 shows an increase of between 430,000 and 12,568,000 km^2^ depending on assumptions about population and economic growth and on estimates of contemporary urban land cover (Table 3). The primary reason for the large variance in the forecasts is the more than tenfold difference in areal estimates of contemporary urban land cover. The areal extent of urban land cover generated by GLC00, MODIS, and GRUMP are 308,007, 726,943, and 3,524,109 square kilometers, respectively [15]. Using SRES scenario B2, our forecasts show additional urban land area between 587,000 and 7,619,000 km^2^ by 2030. The highest estimates were generated using the GRUMP data set as the baseline for contemporary urban land extent. This data set has been shown to generate considerably higher global estimates of urban land cover than other data sets, by nearly five times the MODIS estimates, and ten times greater than the GLC00 estimates [15].

**Table 3:** Forecasts of Additional Urban Land Area by 2030 Using SRES Scenarios†.

Baselinedata set	Baseline urban extent (km^2^)	Additional Urban Land Area by 2030 (km^2^)			
		A1	A2	B1	B2
MODIS 2001‡	726,943	2,255,576	1,165,785	1,913,273	1,526,805
GRUMP 2000	3,524,108	12,568,323	5,734,517	9,818,872	7,619,054
GLC00 2000	307,575	857,528	429,865	719,188	586,177

† SRES Scenarios derived from http://sres.ciesin.columbia.edu/final_data.html.

‡ Based on MOD12Q1 V004 Land Cover Map (http://duckwater.bu.edu/lc/mod12q1.html).

## Discussion

Our model shows that urban land expansion in the fastest growing regions—China, India, and Africa—is driven by different mixes of factors. Annual growth in GDP per capita is related to approximately half of the observed urban land expansion in China but moderate or no expansion in India and Africa. Instead, urban land expansion in India and Africa is related more to urban population growth. Rates of urban land expansion are slower than in high income countries than in low income countries, and increasingly related to GDP growth. In North America, population growth contributes to urban land expansion more than it does in Europe. Much of the observed variation in urban land expansion was not captured by the model. This likely relates to a variety of factors which are difficult to observe comprehensively at the global level including international capital flows, the informal economy, land use policy, and generalized transport costs [20].

Although demographic and economic factors capture a fair amount of urban land expansion in China and India, much of the observed expansion in other regions cannot be accounted for by the explanatory variables of the model. The idiosyncratic nature of the world's urban areas suggests a long list of additional factors that may interact with the fundamentals of population and economic growth in determining urban expansion. Most of these cannot feasibly be gathered globally but four in particular merit further examination. First, the role of international capital, be it foreign direct investment, overseas development assistance, or other types of financial instrument, is key in driving development and especially urban expansion in developing country cities and is excluded from the analysis. Second, in Africa, India, and China, the informal sector forms a substantial portion of the overall economic activity. On average, the informal sector accounts for 44% and 35% of the GNP in Africa and Asia, compared to only 12% of the GNP in OECD countries [20]. Third, land use policies vary significantly between and within metropolitan areas and they distort the fundamental economic dynamics in market and non-market economies alike. For example, all land in China is officially owned by the state, and city officials can lease land through auction or negotiation. Although there is an emerging urban land market, municipal governments have the power to transfer land and establish economic development zones. Urban growth is driven, at least in part, by the economic incentives of local officials to increase their revenue by obtaining rural land and transferring land use rights to developers [21]. Finally, the generalized price of transportation (monetary cost and time cost) also drives the spatial patterns of urban expansion. The transportation of people and goods has generally become less expensive over time with a tendency to promote higher rates of urban expansion. However, the localized nature of this global trend varies based on the proportion of residents with access to motorized transport, the price of fuel, and the spatial distribution of activity centers within the region. Clearly, many factors drive urban expansion at different locations through space and over time. Therefore, the parsimony of this model as well as its scale limits its success.

Our results show that urban areas in low elevation coastal zones are growing faster than elsewhere. With nearly two-thirds of urban areas with populations greater than 5 million located in low-elevation coastal zones, coping with climate change in these rapidly growing coastal urban settlements will require a combination of strategies, including adaptation and mitigation measures such as migration and modification of existing urban space [22]. Inadequate responses to protecting coastal urban areas would be devastating to the economies and infrastructure of 13 percent of the world's urban population.

Our forecasts of global urban land cover for 2030 show a large spread, with a nearly 30-fold range in the estimates (Table 3). The range of the forecasts is largely due to the range of estimates of contemporary urban land cover. On the low end, the forecast of 430,000 km^2^ of new urban land by 2030—an area about the size of Iraq—is generated with the A2 storyline using the GLC00 data set, which is one of the more conservative global estimates of urban land cover. Under this scenario, both population and economic growth rates in the next two decades will need to decline and become lower than current rates of growth. Under the UN low population growth scenario, global population in 2050 will be 8 billion. This is a less likely scenario given that world population is currently 6.88 billion and expected to reach 7 billion by 2011. Similarly, the high end forecast of 12,568,000 km^2^ of new urban land by 2030—an area about the size of the United States and Argentina combined—is generated by using the GRUMP data set with an A1 storyline, a scenario that is also unlikely unless population and economic growth rates both significantly increase.

The more likely forecast of new urban extent is the one generated with the MODIS estimate of contemporary urban land cover using the B2 scenario. The B2 scenario assumes intermediate levels of economic development and continued population increase, albeit at a slower rate than in the A2 scenario. Of the three estimates of contemporary urban land cover, the MODIS-derived estimate is the most up-to-date and internally consistent. Using this combination, our forecast shows an increase of 1,527,000 km^2^ of new urban land area by 2030, an area nearly equal to that of the country of Mongolia. Although there is large uncertainty surrounding the range of population growth estimates, our results show that it is not only population growth that drives urban land expansion. Indeed, for many fast growing regions, population growth explains only a small fraction of the urban land expansion. Other factors such as economic growth, the informal economy, land use policies, and foreign investment will also affect the growth of urban areas.

The strength of the meta-analysis lies in its ability to pool results from individual case studies to develop a generalization of global patterns of urban land expansion. Nonetheless, like all meta-analyses, this study is not without its limitations. First, one source of uncertainty in this study is its use of published materials and only those from English-languages sources. A second limitation of the study is the use of national-level metrics such as GDP to examine a local urban phenomenon. Clearly, a national GDP does not reflect the variation in experiences and processes across multiple urban areas in a single country. Whereas there are databases of estimates of city-level population growth rates, there is no such database of city-level income or GDP. A third limitation of the study is the variation in the image processing techniques used to map urban expansion. Although there are numerous algorithms to identify land-use and land- cover change, there is no consensus as to a “best” technique. The type of change detection method employed will depend largely on data availability, the nature of the landscape under consideration, and the types of urban changes occurring (e.g., increase in urban density versus increase in total urban extent). However, it is important to note that there was limited variation in the types of satellite data used in the studies. This is due to the nature of urban expansion: only moderate (<30 m) to high resolution (<10 m) satellite imagery can accurately identify urban growth. Consequently, a majority of the studies in the meta-analysis used data from the NASA Landsat satellite due to its spatial resolution and its long observational record starting in 1972. Commercial satellite data for land use studies have only been available since 2000. Finally, the collection of urban areas in the meta-study is neither a random nor representative sample of the world's urban settlements. For example, both the largest and smallest cities are underrepresented in the meta-study. Such biases can influence model parameters and projections.

Despite these limitations, the meta-analysis shows four trends that have implications for climate change adaptation, biodiversity, and human well-being. First, the total urban area as reported by the meta-analysis case studies quadrupled over the thirty years while urban population at national levels doubled. Although the meta-analysis does not include all urban areas worldwide, it provides a snapshot of patterns and rates of urban land expansion for 292 case study locations, and the results show that urban areas are expanding faster than urban population growth. Second, urban land expansion is growing faster in low elevation coastal zones than in other areas. This is likely to put millions of people at risk to climate change impacts such as storm surges and sea level rise. Third, rates of urban land expansion near protected areas are as high as in other regions. This will challenge conservation strategies because future urban expansion is expected to be both significant in total area extent and also as likely to occur near protected areas as other regions. Fourth, urban population growth and GDP explain only a percentage of urban land expansion; non-demographic factors and economic dynamics not captured by GDP also play a large role. Although global urban population is expected to increase to 5 billion by 2030 from 3.1 billion in 2010, the results indicate that many non-demographic factors, including land use policies, transportation costs, and income will shape the size of global urban extent in the coming decades.

## Supporting Information

Figure S1 **Regional breakdown of papers included in meta-analysis (number of papers in parentheses).**
(TIF)

Figure S2 **Regional breakdown of all case studies across all papers and locations included in meta-analysis (number of case studies in parentheses).**
(TIF)

Figure S3 **Regional breakdown of locations included in meta-analysis (number of locations in parentheses).**
(TIF)

Figure S4 **Regional breakdown of the proportion of the world's largest agglomerations captured in meta-analysis (Source: authors' calculations and UN WUP 2007).**
(TIF)

Table S1 **Journals included in meta-analysis.**
(DOCX)

Text S1 **Summary of studies.**
(DOCX)

Text S2 **Studies included in meta-analysis.**
(DOCX)

Text S3 **Meta-analysis methodology.**
(DOCX)

## References

[1] Foley JA, DeFries R, Asner GP, Barford C, Bonan G (2005). Global consequences of land use.. Science.

[2] Grimm NB, Faeth SH, Golubiewski NE, Redman CL, Wu JG (2008). Global change and the ecology of cities.. Science.

[3] Hahs AK, McDonnell MJ, McCarthy MA, Vesk PA, Corlett RT (2009). A global synthesis of plant extinction rates in urban areas.. Ecology Letters.

[4] Seto KC, Kaufmann RK, Woodcock CE (2000). Landsat reveals China's farmland reserves, but they're vanishing fast.. Nature.

[5] Radeloff VC, Stewart SI, Hawbaker TJ, Gimmi U, Pidgeon AM (2010). Housing growth in and near United States protected areas limits their conservation value.. Proceedings of the National Academy of Sciences of the United States of America.

[6] Arnfield AJ (2003). Two decades of urban climate research: A review of turbulence, exchanges of energy and water, and the urban heat island.. International Journal of Climatology.

[7] Rosenfeld D (2000). Suppression of rain and snow by urban and industrial air pollution.. Science.

[8] Shepherd JM, Pierce H, Negri AJ (2002). Rainfall modification by major urban areas: Observations from spaceborne rain radar on the TRMM satellite.. Journal of Applied Meteorology.

[9] Bento AM, Cropper ML, Mobarak AM, Vinha K (2005). The effects of urban spatial structure on travel demand in the United States.. Review of Economics and Statistics.

[10] Brownstone D, Golob TF (2009). The impact of residential density on vehicle usage and energy consumption.. Journal of Urban Economics.

[11] Vance C, Hedel R (2007). The impact of urban form on automobile travel: disentangling causation from correlation.. Transportation.

[12] National Research Council (2009). Driving and the built environment: The effects of compact development on motorized travel, energy use, and CO2 emissions.. Transportation Research Board.

[13] Akbari H, Menon S, Rosenfeld A (2009). Global cooling: increasing world-wide urban albedos to offset CO2.. Climatic Change.

[14] Dodman D (2009). Blaming cities for climate change? An analysis of urban greenhouse gas emissions inventories.. Environment and Urbanization.

[15] Potere D, Schneider A (2007). A critical look at representations of urban areas in global maps.. GeoJournal.

[16] National Bureau of Statistics (various years) China Statistical Yearbook..

[17] Center for International Earth Science Information Network (CIESIN) (2002). Country-level Population and Downscaled Projections based on the B2 Scenario, 1990-2100, [digital version]..

[18] United Nations (UN) (2008). World Urbanization Prospects: The 2007 Revision..

[19] Nicholls RJ, Cazenave A (2010). Sea-level rise and its impact on coastal zones.. Science.

[20] Gerxhani K (2004). The informal sector in developed and less developed countries: A literature survey.. Public Choice.

[21] Perlstein A, Seto KC (In review) China' Boom Heads Inland: Understanding Planning, Growth, and Environmental Change in a Central Chinese City..

[22] McGranahan G, Balk D, Anderson B (2007). The rising tide: assessing the risks of climate change and human settlements in low elevation coastal zones.. Environment and Urbanization.

