# Supplementary figures and images for: A Meta-Analysis of Global Urban Land Expansion
Source: PLoS One. 2011 Aug 18;6(8):e23777. doi: 10.1371/journal.pone.0023777 (PMC3158103; doi:10.1371/journal.pone.0023777)

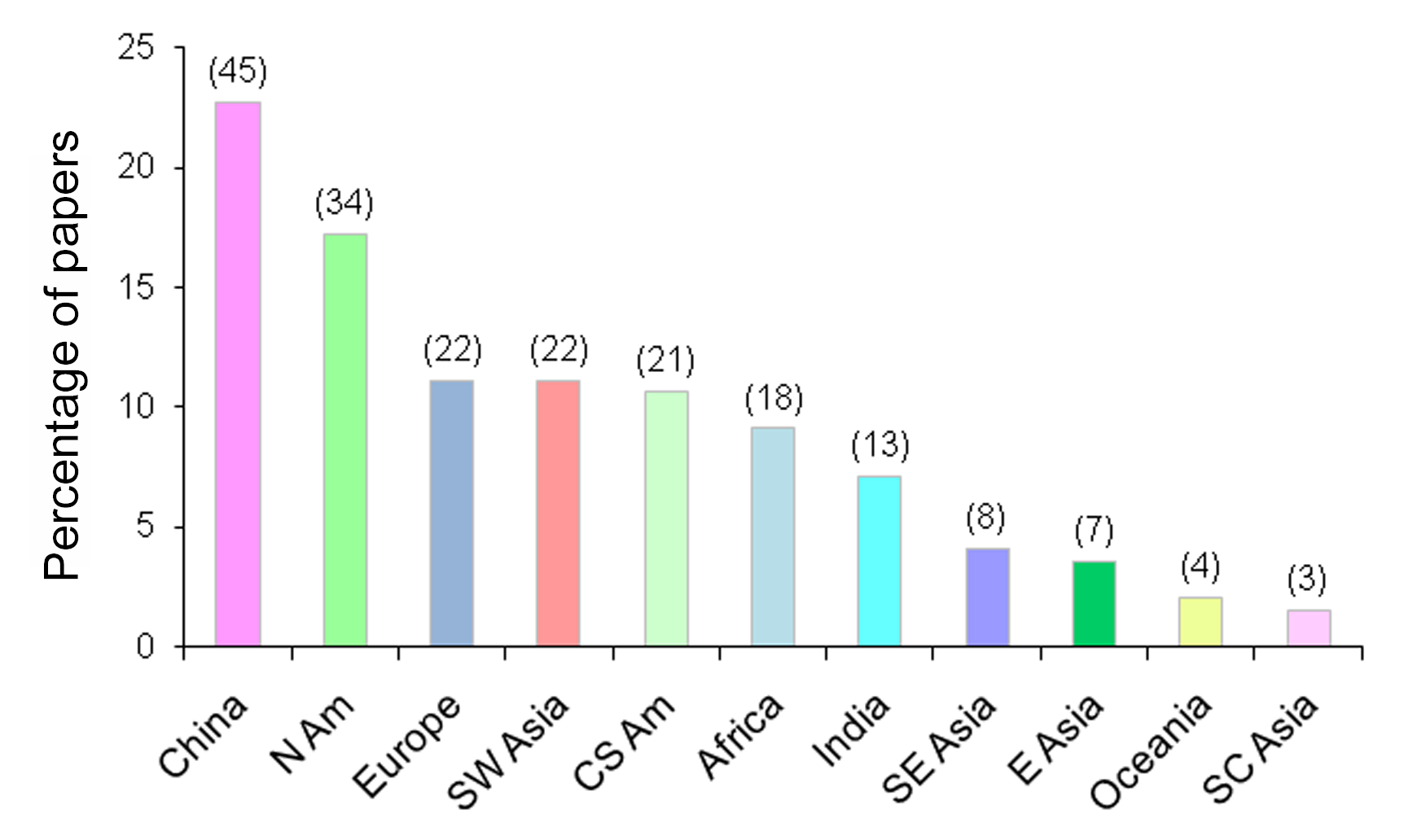

Supplement: Figure S1 — Regional breakdown of papers included in meta-analysis (number of papers in parentheses). (TIF) [file pone.0023777.s001.tif]

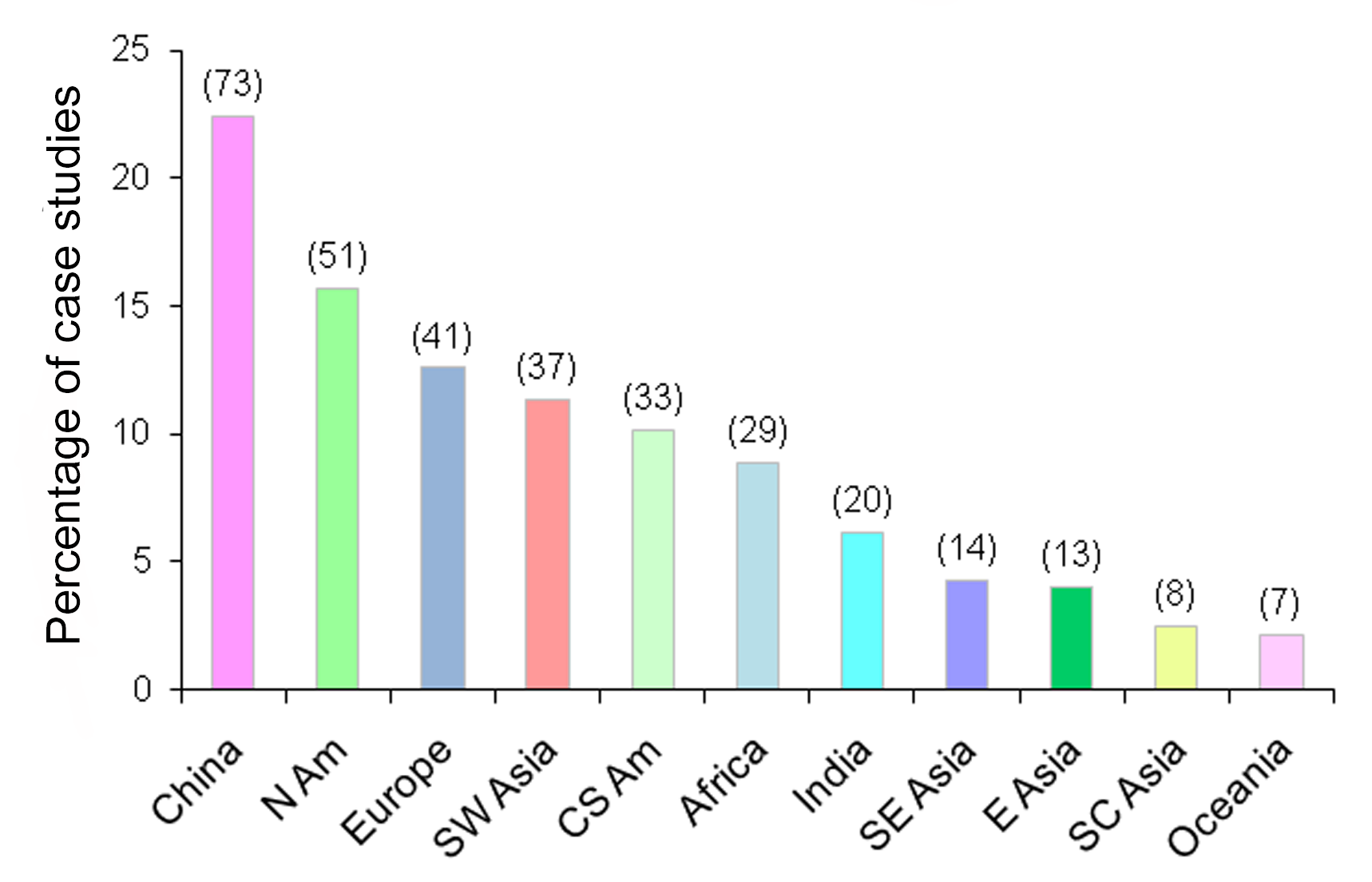

Supplement: Figure S2 — Regional breakdown of all case studies across all papers and locations included in meta-analysis (number of case studies in parentheses). (TIF) [file pone.0023777.s002.tif]

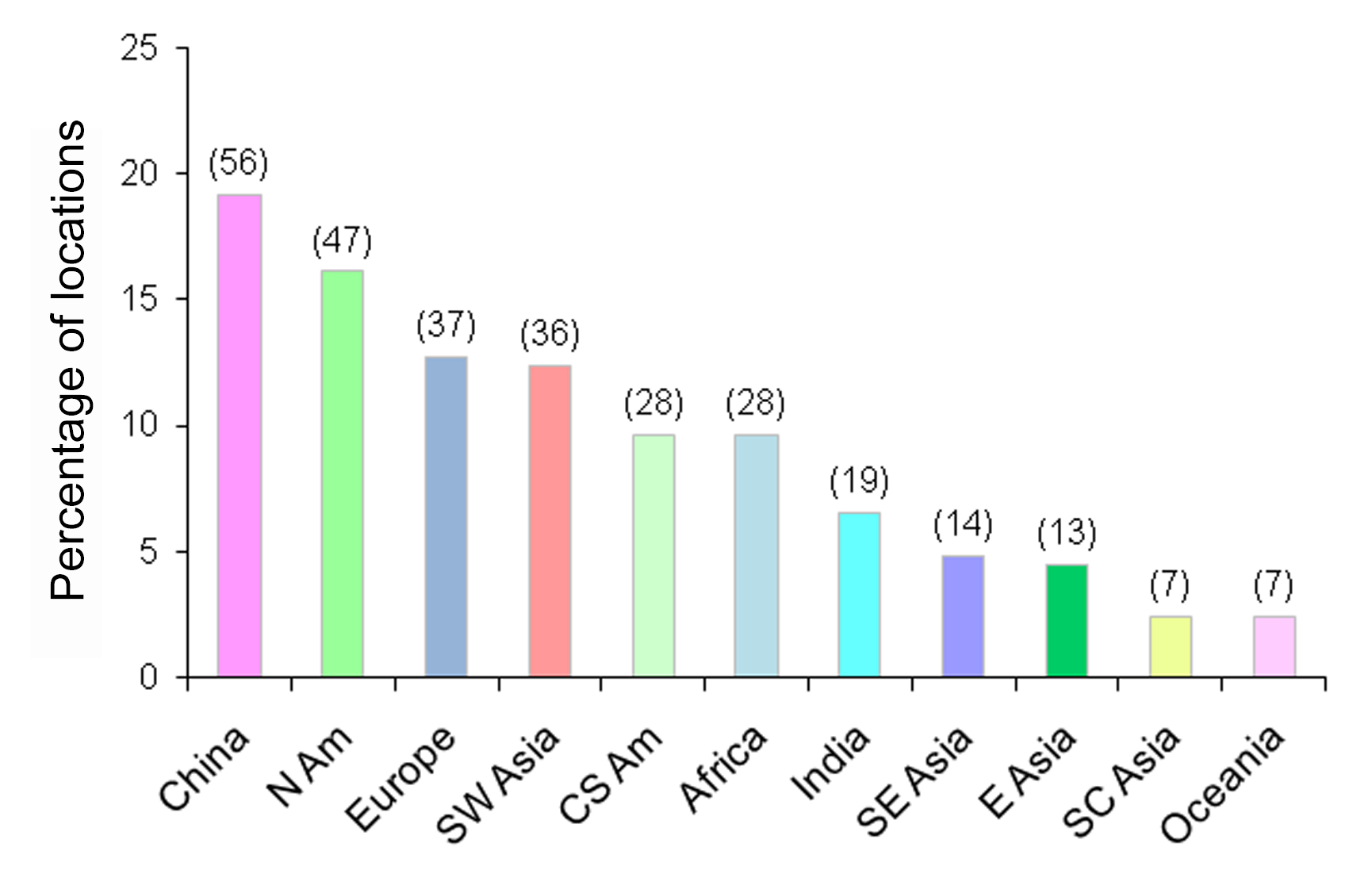

Supplement: Figure S3 — Regional breakdown of locations included in meta-analysis (number of locations in parentheses). (TIF) [file pone.0023777.s003.tif]

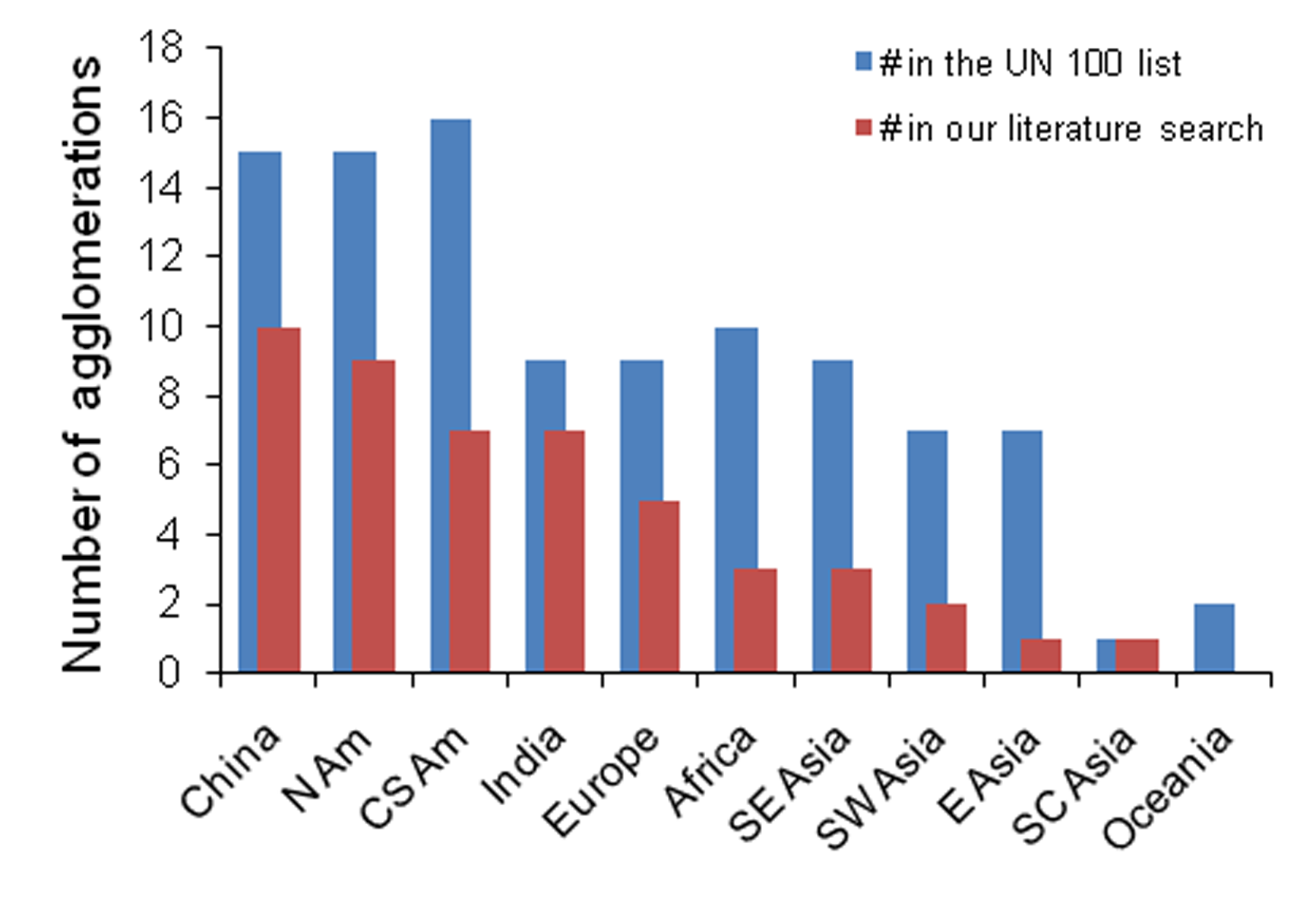

Supplement: Figure S4 — Regional breakdown of the proportion of the world's largest agglomerations captured in meta-analysis (Source: authors' calculations and UN WUP 2007). (TIF) [file pone.0023777.s004.tif]
