# Supplementary material for: A Meta-Analysis of Global Urban Land Expansion
Source: PLoS One. 2011 Aug 18;6(8):e23777. doi: 10.1371/journal.pone.0023777 (PMC3158103; doi:10.1371/journal.pone.0023777)
Supplement: Table S1 — Journals included in meta-analysis. (DOCX) [file pone.0023777.s005.docx]

**Table S1. Journals from which 4 or more papers are included in the meta-analysis**

| **Journal** | **Number of papers** |
| --- | --- |
| International Journal of Remote Sensing | 20 |
| Landscape and Urban Planning | 14 |
| Environmental Monitoring and Assessment | 11 |
| Journal of Environmental Management | 7 |
| Environmental Management | 6 |
| Photogrammetric Engineering and Remote Sensing | 6 |
| Land Degradation & Development | 5 |
| Remote Sensing of Environment | 5 |
| Sensors | 5 |
| Catena | 4 |
| Environment and Planning B | 4 |
| International Journal of Sustainable Development and World Ecology | 4 |
| Land Use Policy | 4 |
| Landscape Ecology | 4 |
