# Supplementary material for: A Meta-Analysis of Global Urban Land Expansion
Source: PLoS One. 2011 Aug 18;6(8):e23777. doi: 10.1371/journal.pone.0023777 (PMC3158103; doi:10.1371/journal.pone.0023777)
Supplement: Text S1 — Summary of studies. (DOCX) [file pone.0023777.s006.docx]

**A Meta-Analysis of Global Urban Land Expansion**

**Text S1. Summary of studies**

**Full search strings for literature search**

**Common Keywords to all regions**

Topic=(spot OR landsat OR satellite OR "remote sensing" OR "remotely sensed" OR IRS OR MODIS OR AVHRR OR aerial OR area OR map*) AND Topic=("land use" OR landuse OR "land cover" OR landcover OR urban* OR city OR cities OR agri* OR arable OR built* OR metropol* OR deforest* OR forest*) AND Topic=(change OR expansion OR growth OR encroach* OR modif*) AND Topic=(*GEOGRAPHIC LOCATION*)

Refined by: Document Type=( ARTICLE OR ABSTRACT OR LETTER OR EDITORIAL OR REVIEW ) AND Languages=( ENGLISH )

**For each *GEOGRAPHIC LOCATION* the following keyword sequences are used by region**

**Africa:**

Africa* OR Egypt OR Alexandria OR iskandariyah OR cairo OR libya OR Tripoli OR tarabulus OR Algeria OR Algiers OR jazair OR tunis* OR morocco OR rabat OR Casablanca OR beida OR Mauritania OR Nouakchott OR Mali OR Niger OR Niamey OR burkina OR Ouagadougou OR guinea OR Senegal OR Dakar OR Banjul OR Bissau OR Conakry OR “sierra leone” OR Freetown OR Liberia OR Monrovia OR “d’ivoire” OR Abidjan OR Yamoussoukro OR Ghana OR accra OR togo OR lome OR Benin OR “porto-novo” OR Abuja OR Ibadan OR lagos OR Chad OR djamena OR Cameroon OR yaounde OR Malabo OR Bangui OR sudan OR khartoum OR khurtum OR Eritrea OR asmara OR Djibouti OR adis OR Ethiopia OR Somalia OR Mogadishu OR muqdisho OR Kenya OR Nairobi OR Uganda OR kampala OR Mombasa OR "sao tome" OR Gabon OR Libreville OR congo OR Kinshasa OR Brazzaville OR Bujumbura OR Burundi OR Tanzania OR Dodoma OR "dar es salaam" OR "cape verde" OR praia OR canary OR Malabo OR azores OR Angola OR Luanda OR Lubumbashi OR Malawi OR Lilongwe OR zambia OR Lusaka OR Mozambique OR Maputo OR Swaziland OR Mbabane OR Zimbabwe OR Harare OR Namibia OR Windhoek OR Botswana OR Gaborone OR Johannesburg OR Pretoria OR Durban OR Lesotho OR maseru OR madagaskar OR Antananarivo OR Seychelles

**China:**

China OR Chinese OR *(all Chinese provinces)* OR "hong kong" OR macau OR macao OR Beijing OR Peking OR Shanghai OR Shenyang OR Harbin OR Wuhan OR Guangzhou OR Chengdu OR Chongqing OR Kunming OR Urumqi OR Ürümqi

**East Asia:**

"eastasia" OR "east asia" OR japan OR korea OR Taiwan OR tokyo OR yokohama OR osaka OR kobe OR hiroshima OR nagazaki OR nagoya OR sapporo OR kyoto OR fukuoka OR kawasaki OR saitama OR sendai OR Kitakyushu OR seoul OR busan OR pusan OR daegu OR incheon OR gwangju OR daejeon OR ulsan OR taipei OR pyongyang OR "pyong yang"

**Europe:**

europe* OR norway OR sweden OR finland OR denmark OR scandinav* OR oslo OR bergen OR stockholm OR helsinki OR Copenhagen OR Baltic OR russia* OR siberia* OR soviet OR kamch* OR mosc* OR aral OR baikal OR ural OR Estonia* OR Tallinn OR Latvia* OR riga OR Lithuania* OR Vilnius OR minsk OR Belarus* OR kyiv OR kiev OR Kharkov OR ukrain* OR Chisinau OR moldova* OR Warszawa OR Warsaw OR Poland OR Romania* OR Bucharest OR bucuresti OR hungar* OR Budapest OR slovakia* OR Bratislava OR Czech OR praha OR Prague OR Ljubljana OR slovenia* OR Zagreb OR croatia* OR Sarajevo OR bosnia* OR belgrad OR Beograd OR Serbia* OR Yugoslavia* OR Pristine OR prizren OR Kosovo* OR skopje OR Macedonia* OR podgorica OR montenegr* OR tiran* OR albani* OR Bulgaria* OR sofia OR sofiya OR Greece OR Greek OR Athens OR athina OR crete OR Aegean OR Balkan OR adriat* OR Italy OR rome OR Milan* OR sicily OR sicilia OR sardinia OR Monaco OR Austria OR Vienna OR malta OR Switzerland OR berne OR Zurich OR Vaduz OR Liechtenstein OR munich OR germany OR berlin OR hamburg OR Frankfurt OR Luxemb* OR German* OR Amsterdam OR Rotterdam OR Netherlands OR Belgium OR Belgian OR Brussels OR paris OR france OR french OR Corsica OR Andorra OR Barcelona OR Madrid OR spain OR sevill* OR Zaragoza OR Saragossa OR Ibiza OR mallorca OR Minorca OR gibraltar OR Portugal OR porto OR lisboa OR Lisbon OR Ireland OR irish OR Scotland OR Scottish OR england OR Britain OR british OR "united kingdom" OR UK OR Dublin OR Belfast OR edinburgh OR Glasgow OR Iceland OR reykjavik

**India:**

India* OR *(all states in India)* OR Delhi OR Chennai OR Madras OR Bangalore OR Bengaluru OR Bombay OR Mumbai OR Kolkata OR Calcutta OR Hyderabad OR Ahmedabad

**North America:**

"north america" OR "northern america" OR "united states" OR USA OR canada OR canadian OR American OR US OR *(all US states)* OR *(all Canadian provinces)* OR "san francisco" OR sacramento OR "los angeles" OR "san diego" OR seattle OR vancouver OR "salt lake city" OR phoenix OR tucson OR denver OR albequerque OR "kansas city" OR "st louis" OR chicago OR tulsa OR houston OR dallas OR austin OR indianapolis OR cleveland OR cincinnati OR nashville OR Memphis OR philadephia OR boston OR "new york" OR miami OR atlanta OR charlotte OR "new orleans" OR detroit OR minneapolis OR Milwaukee OR edmonton OR winnipeg OR quebec OR montreal OR toronto OR Ottawa OR Labrador OR "nova scotia" OR "prince edward"

**Oceania:**

Oceania* OR austral* OR "new zealand" OR tasm* OR melbourne OR sydney OR wellington OR Auckland OR timor OR fiji OR polynes* OR micrones* OR "new guinea" OR "new caledonia" OR solomon OR guam OR samoa* OR "pacific islands" OR "pacific island"

**Central and South America:**

southamerica* OR centralamerica* OR latinamerica* OR "meso america" OR "meso american" OR mesoamerica* OR "south america" OR "central america" OR "latin america" OR "south american" OR "central american" OR "latin american" OR americas OR amazon* OR argentin* OR "buenos aires" OR chile* OR santiago OR uruguay* OR montevideo OR paraguay* OR asuncion OR bolivia* OR "la paz" OR peru* OR lima OR equador OR quito OR colombia* OR bogota OR venezuela* OR caracas OR guyana* OR Georgetown OR surinam* OR paramaribo OR Guiana* OR cayenne OR brazil* OR brasil* OR caribbean OR trinidad OR "saint lucia" OR "saint-lucia" OR "puerto rico" OR "puerto rican" OR Antilles OR martinique OR barbados OR guadeloupe OR haiti* OR dominic* OR jamaica* OR caicos OR bahama* OR cuba* OR havana OR Habana OR mexico OR mexica* OR baja OR belize* OR Belmopan OR guatemal* OR salvador* OR honduras OR Tegucigalpa OR nicaragua* OR managua OR "costa rica" OR "costa rican" OR panama*

**South and Central Asia:**

"central asia" OR "central asian" OR "turkic republic" OR "turkic republics" OR turkmen* OR taji* OR kazakh* OR uzbek* OR kyrgyz* OR mongol* OR ulan OR Ashgabat OR Bishkek OR Almaty OR Astana OR Tashkent OR Toshkent OR Dushanbe OR afghan* OR kabul OR herat OR Kandahar OR Iran* OR Persia* OR Teh* OR Tah* OR Caucas* OR Azer* OR Armenia* OR Georgia* OR Caspian OR Baku OR Tbilisi OR Yerevan

**Southeast Asia:**

indochina OR "southeast asia" OR "south-east asia" OR "southeastern asia" OR "south-eastern asia" OR thailand OR vietnam OR laos OR lao OR Cambodia OR Philippines OR borneo OR malaysia OR indonesia OR brunei OR Singapore OR Jakarta OR java OR bali OR Sulawesi OR kalimantan OR Maluku OR tenggara OR Sumatra OR papua OR Yangon OR Rangoon OR Bangkok OR "kuala lumpur" OR Hanoi OR "ha noi" OR Haiphong OR "hai phong" OR "Ho Chi Minh" OR "Phnom Penh"

**Southwest Asia:**

syria OR aleppo OR damascus OR halep OR sam OR halab OR levant* OR lebanon OR beirut OR israel* OR jordan* OR amman OR haifa OR "tel aviv" OR jerusalem OR kudus OR "shatt-ul-arab" OR shattularab OR iraq* OR kuwait* OR "persian gulf" OR "arabian gulf" OR "gulf state" OR bahrain OR "united arab emirates" OR "abu dhabi" OR dubai OR qatar* OR doha OR kuwayt* OR oman* OR baghdad OR basra OR basrah OR yemen* OR adan OR aden OR sana* OR saudi OR riyad* OR Jeddah OR cyprus OR cypriot OR nicosia OR lefkos* OR "east mediterranean" OR "eastern mediterranean"

**Summary of studies**

The meta-analysis case studies include 67 countries, with 19% and 16% of the locations in China and North America, respectively (Figure S3). The meta-analysis case studies capture only a portion of the world's largest urban agglomerations circa 2007; 48 of the world's 100 currently largest urban areas have been studied - with findings in peer reviewed journals - up to date (Figure S4).

*International Journal of Remote Sensing* was the journal with the most published studies in our meta-analysis (20 studies total - approximately 7.5% of the total number of papers examined in this meta-analysis - 266). *Landscape and Urban Planning* and *Environmental Monitoring and Assessment* follow with 5.3% and 4.1% of the studies respectively. A list of the journals from which 4 or more papers met all our criteria, is given in Table S1. We observe a high degree of dispersion of relevant information across a variety of journals - the papers in this meta-analysis are published in a total of 78 journals.
