# Supplementary material for: A Meta-Analysis of Global Urban Land Expansion
Source: PLoS One. 2011 Aug 18;6(8):e23777. doi: 10.1371/journal.pone.0023777 (PMC3158103; doi:10.1371/journal.pone.0023777)
Supplement: Text S2 — Studies included in meta-analysis. (DOCX) [file pone.0023777.s007.docx]

**A Meta-Analysis of Global Urban Land Expansion**

**Text S2. Studies included in meta-analysis**

1. Abdullah SA, Nakagoshi N (2006) Changes in landscape spatial pattern in the highly developing state of Selangor, peninsular Malaysia. Landscape and Urban Planning 77: 263-275.

2. Acosta A, Carranza ML, Giancola M (2005) Landscape change and ecosystem classification in a municipal district of a small city (Isernia, Central Italy). Environmental Monitoring and Assessment 108: 323-335.

3. Aguayo MI, Wiegand T, Azocar GD, Wiegand K, Vega CE (2007) Revealing the driving forces of mid-cities urban growth patterns using spatial modeling: a case study of Los Angeles, Chile. Ecology and Society 12.

4. Ahqvist O (2008) Extending post-classification change detection using semantic similarity metrics to overcome class heterogeneity: A study of 1992 and 2001 US National Land Cover Database changes. Remote Sensing of Environment 112: 1226-1241.

5. Al Rawashdeh S, Saleh B (2006) Satellite monitoring of urban spatial growth in the Amman area, Jordan. Journal of Urban Planning and Development-ASCE 132: 211-216.

6. Al-Bakri JT, Taylor JC, Brewer TR (2001) Monitoring land use change in the Badia transition zone in Jordan using aerial photography and satellite imagery. Geographical Journal 167: 248-262.

7. Alberti M, Weeks R, Coe S (2004) Urban land-cover change analysis in Central Puget Sound. Photogrammetric Engineering and Remote Sensing 70: 1043-1052.

8. Alphan H (2003) Land-use change and urbanization of Adana, Turkey. Land Degradation & Development 14: 575-586.

9. Alphan H, Yilmaz KT (2005) Monitoring environmental changes in the Mediterranean coastal landscape: The case of Cukurova, Turkey. Environmental Management 35: 607-619.

10. Alshuwaikhat H, Aina Y, Rahman SM (2006) Integration of urban growth management and strategic environmental assessment to ensure sustainable urban development: The Case of Arabian Gulf Cities. International journal of sustainable development and planning 1: 203-213.

11. Angel S, Sheppard SC, Civco DL, Buckley R, Chabaeva A, et al. (2005) The Dynamics of Global Urban Expansion. Washington D.C.: The World Bank.

12. Ayad YA (2005) Remote sensing and GIS in modeling visual landscape change: a case study of the northwestern arid coast of Egypt. Landscape and Urban Planning 73: 307-325.

13. Azocar G, Romero H, Sanhueza R, Vega C, Aguayo M, et al. (2007) Urbanization patterns and their impacts on social restructuring of urban space in Chilean mid-cities: The case of Los Angeles, Central Chile. Land Use Policy 24: 199-211.

14. Baessler C, Klotz S (2006) Effects of changes in agricultural land-use on landscape structure and arable weed vegetation over the last 50 years. Agriculture Ecosystems & Environment 115: 43-50.

15. Baldyga TJ, Miller SN, Driese KL, Gichaba CM (2008) Assessing land cover change in Kenya's Mau Forest region using remotely sensed data. African Journal of Ecology 46: 46-54.

16. Barr M, Graham D, McGuire J (1999) Changes in Manaus in our lifetime as viewed by KidSat. IEEE Transactions on Geoscience and Remote Sensing 37: 1818-1826.

17. Baskent EZ, Kadiogullari AI (2007) Spatial and temporal dynamics of land use pattern in Turkey: A case study in Inegol. Landscape and Urban Planning 81: 316-327.

18. Bektas F, Goksel C (2005) Remote sensing and GIS integration for land cover analysis, a case study: Bozcaada Island. Water Science and Technology 51: 239-244.

19. Bellot J, Bonet A, Pena J, Sanchez JR (2007) Human impacts on land cover and water balances in a coastal Mediterranean county. Environmental Management 39: 412-422.

20. Bender O, Boehmer HJ, Jens D, Schumacher KP (2005) Analysis of land-use change in a sector of Upper Franconia (Bavaria, Germany) since 1850 using land register records. Landscape Ecology 20: 149-163.

21. Benguigui L, Blumenfeld-Lieberthal E, Czarnanski D (2006) The dynamics of the Tel Aviv morphology. Environment and Planning B-Planning & Design 33: 269-284.

22. Bergen KM, Zhao T, Kharuk V, Blam Y, Brown DG, et al. (2008) Changing regimes: Forested land cover dynamics in Central Siberia 1974 to 2001. Photogrammetric Engineering and Remote Sensing 74: 787-798.

23. Bilby RE, Mollot LA (2008) Effect of changing land use patterns on the distribution of coho salmon (Oncorhynchus kisutch) in the Puget Sound region. Canadian Journal of Fisheries and Aquatic Sciences 65: 2138-2148.

24. Boentje JP, Blinnikov MS (2007) Post-Soviet forest fragmentation and loss in the Green Belt around Moscow, Russia (1991-2001): a remote sensing perspective. Landscape and Urban Planning 82: 208-221.

25. Boyle CA, Lavkulich L, Schreier H, Kiss E (1997) Changes in land cover and subsequent effects on lower fraser basin ecosystems from 1827 to 1990. Environmental Management 21: 185-196.

26. Braimoh AK, Onishi T (2007) Spatial determinants of urban land use change in Lagos, Nigeria. Land Use Policy 24: 502-515.

27. Burnside NG, Smith RF, Waite S (2003) Recent historical land use change on the South Downs, United Kingdom. Environmental Conservation 30: 52-60.

28. Catalan B, Sauri D, Serra P (2008) Urban sprawl in the Mediterranean? Patterns of growth and change in the Barcelona Metropolitan Region 1993-2000. Landscape and Urban Planning 85: 174-184.

29. Cetin M, Musaoglu N, Tanik A (2008) Multitemporal Assessment of Land-Use Change in a Rapidly Urbanizing Coastal Region in Turkey Using Remote Sensing. Environmental Engineering Science 25: 917-928.

30. Chen XW (2002) Using remote sensing and GIS to analyse land cover change and its impacts on regional sustainable development. International Journal of Remote Sensing 23: 107-124.

31. Cheng SJ, Wang RY (2002) An approach for evaluating the hydrological effects of urbanization and its application. Hydrological Processes 16: 1403-1418.

32. Cisternas M, Araneda A, Martinez P, Perez S (2001) Effects of historical land use on sediment yield from a lacustrine watershed in central Chile. Earth Surface Processes and Landforms 26: 63-76.

33. Comber AJ, Birnie RV, Hodgson M (2003) A retrospective analysis of land cover change using a polygon shape index. Global Ecology and Biogeography 12: 207-215.

34. Corona P, Fattorini L, Pompei E (2007) Aerial assessment of landscape net change by means of two-phase network sampling: an application to central Italy. Environmetrics 18: 205-215.

35. DiBari JN (2007) Evaluation of five landscape-level metrics for measuring the effects of urbanization on landscape structure: the case of Tucson, Arizona, USA. Landscape and Urban Planning 79: 308-313.

36. Ding H, Wang RC, Wu JP (2007) Quantifying land use change in Zhejiang coastal region, China using multi-temporal Landsat TM/ETM plus images. Pedosphere 17: 712-720.

37. Dong W, Zhang XL, Wang B, Duan ZL (2007) Expansion of Ürümqi urban area and its spatial differentiation. Science in China Series D-Earth Sciences 50: 159-168.

38. Doygun H, Alphan H (2006) Monitoring urbanization of Iskenderun, Turkey, and its negative implications. Environmental Monitoring and Assessment 114: 145-155.

39. El-Gamily HI, Nasr S, El-Raey M (2001) An assessment of natural and human-induced changes along Hurghada and Ras Abu Soma coastal area, Red Sea, Egypt. International Journal of Remote Sensing 22: 2999-3014.

40. Esbah H (2007) Land use trends during rapid urbanization of the city of Aydin, Turkey. Environmental Management 39: 443-459.

41. Fan FL, Weng QH, Wang YP (2007) Land use and land cover change in Guangzhou, China, from 1998 to 2003, based on Landsat TM/ETM+ imagery. Sensors 7: 1323-1342.

42. Fazal S (2000) Urban expansion and loss of agricultural land - a GIS based study of Saharanpur City, India. Environment and Urbanization 12: 133-149.

43. Fazal S (2001) The need for preserving farmland - A case study from a predominantly agrarian economy (India). Landscape and Urban Planning 55: 1-13.

44. Flint EP, Richards JF (1991) Historical-analysis of changes in land-use and carbon stock of vegetation in South and Southeast Asia. Canadian Journal of Forest Research-Revue Canadienne De Recherche Forestiere 21: 91-110.

45. Fu B, Gulinck H, Masum MZ (1994) Loess erosion in relation to land-use changes in the Ganspoel catchment, Central Belgium. Land Degradation and Rehabilitation 5: 261-270.

46. Galster JC, Pazzaglia FJ, Germanoski D (2008) Measuring the impact of urbanization on channel widths using historic aerial photographs and modern surveys. Journal of the American Water Resources Association 44: 948-960.

47. Garfi G, Bruno DE, Calcaterra D, Parise M (2007) Fan morphodynamics and slope instability in the Mucone River basin (Sila Massif, southern Italy): Significance of weathering and role of land use changes. Catena 69: 181-196.

48. Gomarasca MA, Brivio PA, Pagnoni F, Galli A (1993) One century of and-use changes in the metropolitan area of Milan (Italy). International Journal of Remote Sensing 14: 211-223.

49. Gonzalez OMR (2001) Assessing vegetation and land cover changes in northeastern Puerto Rico: 1978-1995. Caribbean Journal of Science 37: 95-106.

50. Guler M, Yomralioglu T, Reis S (2007) Using Landsat data to determine land use/land cover changes in Samsun, Turkey. Environmental Monitoring and Assessment 127: 155-167.

51. Ha SR, Bae MS (2001) Effects of land use and municipal wastewater treatment changes on stream water quality. Environmental Monitoring and Assessment 70: 135-151.

52. Hashiba H, Kameda K, Sugimura T, Takasaki K (1998) Analysis of landuse change in periphery of Tokyo during last twenty years using the same seasonal Landsat data. Advances in Space Research 22: 681-684.

53. Hathout S (2002) The use of GIS for monitoring and predicting urban growth in East and West St Paul, Winnipeg, Manitoba, Canada. Journal of Environmental Management 66: 229-238.

54. He CY, Okada N, Zhang QF, Shi PJ, Zhang JS (2006) Modeling urban expansion scenarios by coupling cellular automata model and system dynamic model in Beijing, China. Applied Geography 26: 323-345.

55. Henriquez C, Azocar G, Romero H (2006) Monitoring and modeling the urban growth of two mid-sized Chilean cities. Habitat International 30: 945-964.

56. Ho SPS, Lin GCS (2004) Converting land to nonagricultural use in China's coastal provinces - Evidence from Jiangsu. Modern China 30: 81-112.

57. Hu D, Yang GD, Wu Q, Li HQ, Liu XS, et al. (2008) Analyzing land use changes in the metropolitan Jilin City of Northeastern China using remote sensing and GIS. Sensors 8: 5449-5465.

58. Hu HB, Liu WJ, Cao M (2008) Impact of land use and land cover changes on ecosystem services in Menglun, Xishuangbanna, Southwest China. Environmental Monitoring and Assessment 146: 147-156.

59. Ichikawa K, Okubo N, Okubo S, Takeuchi K (2006) Transition of the Satoyama landscape in the urban fringe of the Tokyo metropolitan area from 1880 to 2001. Landscape and Urban Planning 78: 398-410.

60. Ierodiaconou D, Laurenson L, Leblanc M, Stagnitti F, Duff G, et al. (2005) The consequences of land use change on nutrient exports: a regional scale assessment in south-west Victoria, Australia. Journal of Environmental Management 74: 305-316.

61. Jansen LJM, Bagnoli M, Focacci M (2008) Analysis of land-cover/use change dynamics in Manica Province in Mozambique in a period of transition (1990-2004). Forest Ecology and Management 254: 308-326.

62. Jat MK, Garg PK, Khare D (2008) Monitoring and modelling of urban sprawl using remote sensing and GIS techniques. International Journal of Applied Earth Observation and Geoinformation 10: 26-43.

63. Jat MK, Garg PK, Khare D (2008) Modelling of urban growth using spatial analysis techniques: a case study of Ajmer city (India). International Journal of Remote Sensing 29: 543-567.

64. Jazcilevich A, Fuentes V, Jauregui E, Luna E (2000) Simulated urban climate response to historical land use modification in the Basin of Mexico. Climatic Change 44: 515-536.

65. Ji W, Ma J, Twibell RW, Underhill K (2006) Characterizing urban sprawl using multi-stage remote sensing images and landscape metrics. Computers Environment and Urban Systems 30: 861-879.

66. Johnson AKL, Ebert SP, Murray AE (2000) Land cover change and its environmental significance in the Herbert River catchment, north-east Queensland. Australian Geographer 31: 75-86.

67. Jomaa I, Auda Y, Saleh BA, Hamze M, Safi S (2008) Landscape spatial dynamics over 38 years under natural and anthropogenic pressures in Mount Lebanon. Landscape and Urban Planning 87: 67-75.

68. Jusoff K, Senthavy S (2003) Land use change detection using remote sensing and geographical information system (GIS) in Gua Musang district, Kelantan, Malaysia. Journal of Tropical Forest Science 15: 303-312.

69. Kadiogullari AI, Baskent EZ (2008) Spatial and temporal dynamics of land use pattern in Eastern Turkey: a case study in Gumushane. Environmental Monitoring and Assessment 138: 289-303.

70. Kamusoko C, Aniya M (2007) Land use/cover change and landscape fragmentation analysis in the Bindura District, Zimbabwe. Land Degradation & Development 18: 221-233.

71. Khan SD (2005) Urban development and flooding in Houston Texas, inferences from remote sensing data using neural network technique. Environmental Geology 47: 1120-1127.

72. Kilic S, Evrendilek F, Berberoglu S, Demirkesen A (2006) Environmental monitoring of land-use and land-cover changes in a Mediterranean region of Turkey. Environmental Monitoring and Assessment 114: 157-168.

73. Kintz DB, Young KR, Crews-Meyer KA (2006) Implications of land use/land cover change in the buffer zone of a National Park in the Tropical Andes. Environmental Management 38: 238-252.

74. Kreuter UP, Harris HG, Matlock MD, Lacey RE (2001) Change in ecosystem service values in the San Antonio area, Texas. Ecological Economics 39: 333-346.

75. Lawrence WT, Imhoff ML, Kerle N, Stutzer D (2002) Quantifying urban land use and impact on soils in Egypt using diurnal satellite imagery of the Earth surface. International Journal of Remote Sensing 23: 3921-3937.

76. Leao S, Bishop I, Evans D (2004) Simulating urban growth in a developing nation's region using a cellular automata-based model. Journal of Urban Planning and Development-ASCE 130: 145-158.

77. Lein JK, Day KL (2008) Assessing the growth-inducing impact of the Appalachian Development Highway System in southern Ohio: Did policy promote change? Land Use Policy 25: 523-532.

78. Li GL, Chen J, Sun ZY (2007) Non-agricultural land expansion and driving forces: a multi-temporal study of Suzhou, China. International Journal of Sustainable Development and World Ecology 14: 408-420.

79. Li RQ, Dong M, Cui JY, Zhang LL, Cui QG, et al. (2007) Quantification of the impact of land-use changes on ecosystem services: A case study in Pingbian County, China. Environmental Monitoring and Assessment 128: 503-510.

80. Li WF, Wang YL, Peng F, Li GC (2005) Landscape spatial changes associated with rapid urbanization in Shenzhen, China. International Journal of Sustainable Development and World Ecology 12: 314-325.

81. Li X (1998) Measurement of rapid agricultural land loss in the Pearl River Delta with the integration of remote sensing and GIS. Environment and Planning B-Planning & Design 25: 447-461.

82. Li XY, Wang ZM, Song KS, Zhang B, Liu DW, et al. (2007) Assessment for salinized wasteland expansion and land use change using GIS and remote sensing in the west part of Northeast China. Environmental Monitoring and Assessment 131: 421-437.

83. Li Z, Li X, Wang Y, Ma A, Wang J (2004) Land-use change analysis in Yulin prefecture, northwestern China using remote sensing and GIS. International Journal of Remote Sensing 25: 5691-5703.

84. Lin GCS (2004) The Chinese globalizing cities: national centers of globalization and urban transformation. Progress in Planning 61: 143-157.

85. Lin GCS (2007) Reproducing spaces of Chinese urbanisation: New city-based and land-centred urban transformation. Urban Studies 44: 1827-1855.

86. Liu L, Dong X, Wang J (2007) Dynamic analysis of eco-environmental changes based on remote sensing and geographic information system: an example in Longdong region of the Chinese Loess Plateau. Environmental Geology 53: 589-598.

87. Long HL, Tang GP, Li XB, Heilig GK (2007) Socio-economic driving forces of land-use change in Kunshan, the Yangtze River Delta economic area of China. Journal of Environmental Management 83: 351-364.

88. Long HL, Wu XQ, Wang WJ, Dong GH (2008) Analysis of urban-rural land-use change during 1995-2006 and its policy dimensional driving forces in Chongqing, China. Sensors 8: 681-699.

89. Lopez E, Bocco G, Mendoza M, Duhau E (2001) Predicting land-cover and land-use change in the urban fringe - A case in Morelia city, Mexico. Landscape and Urban Planning 55: 271-285.

90. Lozano AV, Vidal CA, Diaz JS (2007) Urban growth (1956-1998) in the metropolitan area of Alacant-Elx (Comunitat Valenciana). Boletin De La Asociacion De Geografos Espanoles: 367-370.

91. Lu Y, Wang J, Wei L, Mo J (2006) Land use change and its impact on values of ecosystem services in the West of Jilin Province. Wuhan University Journal of Natural Sciences 11: 1028-1034.

92. Lunetta RS, Knight JF, Ediriwickrema J, Lyon JG, Worthy LD (2006) Land-cover change detection using multi-temporal MODIS NDVI data. Remote Sensing of Environment 105: 142-154.

93. Madhavan BB, Kubo S, Kurisaki N, Sivakumar T (2001) Appraising the anatomy and spatial growth of the Bangkok Metropolitan area using a vegetation-impervious-soil model through remote sensing. International Journal of Remote Sensing 22: 789-806.

94. Mahiny AS, Gholamalifard M (2007) Dynamic spatial modeling of urban growth through cellular automata in a GIS environment. International Journal of Environmental Research 1: 272-279.

95. Marathianou M, Kosmas C, Gerontidis S, Detsis V (2000) Land-use evolution and degradation in Lesvos (Greece): A historical approach. Land Degradation & Development 11: 63-73.

96. Martin BA, Shao G, Swihart RK, Parker GR, Tang L (2008) Implications of shared edge length between land cover types for landscape quality: the case of Midwestern US, 1940-1998. Landscape Ecology 23: 391-402.

97. McCusker B, Ramudzuli M (2007) Apartheid spatial engineering and land use change in Mankweng, South Africa: 1963-2001. Geographical Journal 173: 56-74.

98. Messina JP, Walsh SJ (2001) 2.5D Morphogenesis: modeling landuse and landcover dynamics in the Ecuadorian Amazon. Plant Ecology 156: 75-88.

99. Moreira F, Rego FC, Ferreira PG (2001) Temporal (1958-1995) pattern of change in a cultural landscape of northwestern Portugal: implications for fire occurrence. Landscape Ecology 16: 557-567.

100. Mundia CN, Aniya A (2006) Dynamics of landuse/cover changes and degradation of Nairobi City, Kenya. Land Degradation & Development 17: 97-108.

101. Murali RM, Vethamony P, Saran AK, Jayakumar S (2006) Change detection studies in coastal zone features of Goa, India by remote sensing. Current Science 91: 816-820.

102. Muriuki GW, Njoka TJ, Reid RS, Nyariki DM (2005) Tsetse control and land-use change in Lambwe valley, south-western Kenya. Agriculture Ecosystems & Environment 106: 99-107.

103. Murthy Y, Raghavswamy V, Pathan SK, Majumdar KL (1991) IRS-1A applications for urban planning. Current Science 61: 243-246.

104. Muttitanon W, Tripathi NK (2005) Land use/land cover changes in the coastal zone of Ban Don Bay, Thailand using Landsat 5 TM data. International Journal of Remote Sensing 26: 2311-2323.

105. Ningal T, Hartemink AE, Bregt AK (2008) Land use change and population growth in the Morobe province of Papua New Guinea between 1975 and 2000. Journal of Environmental Management 87: 117-124.

106. Novak AB, Wang YQ (2004) Effects of suburban sprawl on Rhode Island's forests: A Landsat view from 1972 to 1999. Northeastern Naturalist 11: 67-74.

107. O'Hara CG, King JS, Cartwright JH, King RL (2003) Multitemporal land use and land cover classification of urbanized areas within sensitive coastal environments. IEEE Transactions on Geoscience and Remote Sensing 41: 2005-2014.

108. Ojeda-Revah L, Bocco G, Ezcurra E, Espejel I (2008) Land-cover/use transitions in the binational Tijuana River watershed during a period of rapid industrialization. Applied Vegetation Science 11: 107-116.

109. Otto R, Krusi BO, Kienast F (2007) Degradation of an arid coastal landscape in relation to land use changes in Southern Tenerife (Canary Islands). Journal of Arid Environments 70: 527-539.

110. Pan XZ, Zhao QG (2007) Measurement of urbanization process and the paddy soil loss in Yixing city, China between 1949 and 2000. Catena 69: 65-73.

111. Papastergiadou ES, Retalis A, Apostolakis A, Georgiadis T (2008) Environmental monitoring of spatio-temporal changes using remote sensing and GIS in a Mediterranean wetland of Northern Greece. Water Resources Management 22: 579-594.

112. Pascarella JB, Aide TM, Serrano MI, Zimmerman JK (2000) Land-use history and forest regeneration in the Cayey Mountains, Puerto Rico. Ecosystems 3: 217-228.

113. Pascual JA, Ano C, Valera A, Poyatos M, Sanchez J (2005) Urban growth (1956-1998) and soil degradation in the municipality of Valencia, Spain. Sustainable Use and Management of Soils - Arid and Semiarid Regions 36: 347-354.

114. Pathan SK, Sastry SVC, Dhinwa PS, Rao M, Majumdar KL, et al. (1993) Urban growth trend analysis using GIS techniques - a case study of the Bombay metropolitan region. International Journal of Remote Sensing 14: 3169-3179.

115. Pauchard A, Aguayo M, Pena E, Urrutia R (2006) Multiple effects of urbanization on the biodiversity of developing countries: The case of a fast-growing metropolitan area (Concepcion, Chile). Biological Conservation 127: 272-281.

116. Peng J, Wang YL, Wu JS, Yue J, Zhang YA, et al. (2006) Ecological effects associated with land-use change in China's southwest agricultural landscape. International Journal of Sustainable Development and World Ecology 13: 315-325.

117. Petit C, Scudder T, Lambin E (2001) Quantifying processes of land-cover change by remote sensing: resettlement and rapid land-cover changes in south-eastern Zambia. International Journal of Remote Sensing 22: 3435-3456.

118. Polyakov M, Zhang DW (2008) Property tax policy and land-use change. Land Economics 84: 396-408.

119. Prol-Ledesma RM, Uribe-Alcantara EM, Diaz-Molina O (2002) Use of cartographic data and Landsat TM images to determine land use change in the vicinity of Mexico City. International Journal of Remote Sensing 23: 1927-1933.

120. Qi SZ, Li XY, Duan HP (2007) Oasis land-use change and its environmental impact in Jinta Oasis, arid northwestern China. Environmental Monitoring and Assessment 134: 313-320.

121. Qiu F, Woller KL, Briggs R (2003) Modeling urban population growth from remotely sensed imagery and TIGER GIS road data. Photogrammetric Engineering and Remote Sensing 69: 1031-1042.

122. Quan B, Chen JF, Qiu HL, Romkens MJM, Yang XQ, et al. (2006) Spatial-temporal pattern and driving forces of land use changes in Xiamen. Pedosphere 16: 477-488.

123. Rao BRM, Dwivedi RS, Kushwaha SPS, Bhattacharya SN, Anand JB, et al. (1999) Monitoring the spatial extent of coastal wetlands using ERS-1 SAR data. International Journal of Remote Sensing 20: 2509-2517.

124. Raumann CG, Cablk ME (2008) Change in the forested and developed landscape of the Lake Tahoe basin, California and Nevada, USA, 1940-2002. Forest Ecology and Management 255: 3424-3439.

125. Routray JK, Rath KC, Sahoo NN (1996) Growth, development and planning of Bhubaneswar : A city in eastern India. Cities 13: 79-96.

126. Royer A, Charbonneau L, Bonn F (1988) Urbanization and Landsat MSS albedo change in the Windsor Quebec corridor since 1972. International Journal of Remote Sensing 9: 555-566.

127. Ruiz-Luna A, Berlanga-Robles CA (2003) Land use, land cover changes and coastal lagoon surface reduction associated with urban growth in northwest Mexico. Landscape Ecology 18: 159-171.

128. Saghafian B, Farazjoo H, Bozorgy B, Yazdandoost F (2008) Flood intensification due to changes in land use. Water Resources Management 22: 1051-1067.

129. Satapathy DR, Krupadam RJ, Kumar LP, Wate SR (2007) The application of satellite data for the quantification of mangrove loss and coastal management in the Godavari estuary, East Coast of India. Environmental Monitoring and Assessment 134: 453-469.

130. Schneider A, Seto KC, Webster DR (2005) Urban growth in Chengdu, Western China: application of remote sensing to assess planning and policy outcomes. Environment and Planning B 32: 323-345.

131. Schneider A, Woodcock CE (2008) Compact, dispersed, fragmented, extensive? A comparison of urban growth in twenty-five global cities using remotely sensed data, pattern metrics and census information. Urban Studies 45: 659-692.

132. Sertel E, Findik N, Kaya S, Seker DZ, Samsunlu A (2008) Assessment of landscape changes in the Kizilirmak Delta, Turkey, using remotely sensed data and GIS. Environmental Engineering Science 25: 353-361.

133. Shalaby A, Tateishi R (2007) Remote sensing and GIS for mapping and monitoring land cover and land-use changes in the Northwestern coastal zone of Egypt. Applied Geography 27: 28-41.

134. Shoshany M, Goldshleger N (2002) Land-use and population density changes in Israel - 1950 to 1990: analysis of regional and local trends. Land Use Policy 19: 123-133.

135. Sonmez NK, Sari M (2007) Monitoring land use change in the West Mediterranean region of Turkey: A case study on Antalya-Turkey coast. Fresenius Environmental Bulletin 16: 1325-1330.

136. Sorensen A (2000) Land readjustment and metropolitan growth: an examination of suburban land development and urban sprawl in the Tokyo metropolitan area. Progress in Planning 53: 217-330.

137. Stehman SV, Sohl TL, Loveland TR (2003) Statistical sampling to characterize recent United States land-cover change. Remote Sensing of Environment 86: 517-529.

138. Syam T, Nishide H, Salam AK, Utomo M, Mahi AK, et al. (1997) Land use and cover changes in a hilly area of South Sumatra, Indonesia (from 1970 to 1990). Soil Science and Plant Nutrition 43: 587-599.

139. Symeonakis E, Calvo-Cases A, Arnau-Rosalen E (2007) Land use change and land degradation in southeastern Mediterranean Spain. Environmental Management 40: 80-94.

140. Tang J, Wang L, Zhang S (2005) Investigating landscape pattern and its dynamics in Daqing, China. International Journal of Remote Sensing 26: 2259-2280.

141. Tappan GG, Sall M, Wood EC, Cushing M (2004) Ecoregions and land cover trends in Senegal. Journal of Arid Environments 59: 427-462.

142. Thomlinson JR, Rivera LY (2000) Suburban growth in Luquillo, Puerto Rico: some consequences of development on natural and semi-natural systems. Landscape and Urban Planning 49: 15-23.

143. Thomlinson JR, Serrano MI, Lopez TD, Aide TM, Zimmerman JK (1996) Land-use dynamics in a post-agricultural Puerto Rican landscape (1936-1988). Biotropica 28: 525-536.

144. Thongmanivong S, Fujita Y (2006) Recent land use and livelihood transitions in northern Laos. Mountain Research and Development 26: 237-244.

145. Tian GJ, Yang ZF, Xie Y (2007) Detecting spatiotemporal dynamic landscape patterns using remote sensing and the lacunarity index: a case study of Haikou City, China. Environment and Planning B 34: 556-569.

146. Tole L (2008) Changes in the built vs. non-built environment in a rapidly urbanizing region: A case study of the Greater Toronto Area. Computers Environment and Urban Systems 32: 355-364.

147. Tomlinson RW, Milne RM (2006) Soil carbon stocks and land cover in Northern Ireland from 1939 to 2000. Applied Geography 26: 18-39.

148. Torbick NM, Qi JG, Roloff GJ, Stevenson RJ (2006) Investigating impacts of land-use land cover change on wetlands in the Muskegon River Watershed, Michigan, USA. Wetlands 26: 1103-1113.

149. Vanderstraete T, Goossens R, Ghabour TK (2006) The use of multi-temporal Landsat images for the change detection of the coastal zone near Hurghada, Egypt. International Journal of Remote Sensing 27: 3645-3655.

150. Wang XH, Zheng D, Shen YC (2008) Land use change and its driving forces on the Tibetan Plateau during 1990-2000. Catena 72: 56-66.

151. Wang Y, Moskovits DK (2001) Tracking Fragmentation of Natural Communities and Changes in Land Cover: Applications of Landsat Data for Conservation in an Urban Landscape (Chicago Wilderness). Conservation Biology 15: 835-843.

152. Ward D, Phinn SR, Murray AT (2000) Monitoring growth in rapidly urbanizing areas using remotely sensed data. Professional Geographer 52: 371-386.

153. Weber C, Puissant A (2003) Urbanization pressure and modeling of urban growth: example of the Tunis Metropolitan Area. Remote Sensing of Environment 86: 341-352.

154. Weng Q (2001) A remote sensing-GIS evaluation of urban expansion and its impact on surface temperature in the Zhujiang Delta, China. International Journal of Remote Sensing 22: 1999-2014.

155. Weng YC (2007) Spatiotemporal changes of landscape pattern in response to urbanization. Landscape and Urban Planning 81: 341-353.

156. Wilson JS, Lindsey GH (2005) Socioeconomic correlates and environmental impacts of urban development in a central Indiana landscape. Journal of Urban Planning and Development-ASCE 131: 159-169.

157. Wu Q, Li HQ, Wang RS, Paulussen J, He Y, et al. (2006) Monitoring and predicting land use change in Beijing using remote sensing and GIS. Landscape and Urban Planning 78: 322-333.

158. Wu W, Hall CAS, Scatena FN (2007) Modelling the impact of recent land-cover changes on the 25 stream flows in northeastern Puerto Rico. Hydrological Processes 21: 2944-2956.

159. Wu XQ, Cai YL (2004) Land cover changes and landscape dynamics assessment in lower reaches or Tarim River in China. Chinese Geographical Science 14: 28-33.

160. Xian G, Crane M, McMahon C (2008) Quantifying multi-temporal urban development characteristics in Las Vegas from Landsat and ASTER data. Photogrammetric Engineering and Remote Sensing 74: 473-481.

161. Xian G, Crane M, Su J (2007) An analysis of urban development and its environmental impact on the Tampa Bay watershed. Journal of Environmental Management 85: 965-976.

162. Xiao HL, Weng QH (2007) The impact of land use and land cover changes on land surface temperature in a karst area of China. Journal of Environmental Management 85: 245-257.

163. Xiao JY, Shen YJ, Ge JF, Tateishi R, Tang CY, et al. (2006) Evaluating urban expansion and land use change in Shijiazhuang, China, by using GIS and remote sensing. Landscape and Urban Planning 75: 69-80.

164. Xie YC, Fang CL, Lin GCS, Gong HM, Qiao B (2007) Tempo-spatial patterns of land use changes and urban development in globalizing China: A study of Beijing. Sensors 7: 2881-2906.

165. Xie YC, Mei Y, Tian GJ, Xing XR (2005) Socio-econornic driving forces of arable land conversion: A case study of Wuxian City, China. Global Environmental Change-Human and Policy Dimensions 15: 238-252.

166. Xu C, Liu M, An S, Chen JM, Yan P (2007) Assessing the impact of urbanization on regional net primary productivity in Jiangyin County, China. Journal of Environmental Management 85: 597-606.

167. Xu H, Wang X, Xiao G (2000) A remote sensing and GIS integrated study on urbanization with its impact on arable lands: Fuqing City, Fujian Province, China. Land Degradation & Development 11: 301-314.

168. Xu JC, Ai XH, Deng XQ (2005) Exploring the spatial and temporal dynamics of land use in Xizhuang watershed of Yunnan, southwest China. International Journal of Applied Earth Observation and Geoinformation 7: 299-309.

169. Yagoub MM (2004) Monitoring of urban growth of a desert city through remote sensing: Al-Ain, UAE, between 1976 and 2000. International Journal of Remote Sensing 25: 1063-1076.

170. Yagoub MM, Kolan GR (2006) Monitoring coastal zone land use and land cover changes of Abu Dhabi using remote sensing. Photonirvachak-Journal of the Indian Society of Remote Sensing 34: 57-68.

171. Yang X, Lo CP (2002) Using a time series of satellite imagery to detect land use and land cover changes in the Atlanta, Georgia metropolitan area. International Journal of Remote Sensing 23: 1775-1798.

172. Yang XJ (2002) Satellite monitoring of urban spatial growth in the Atlanta metropolitan area. Photogrammetric Engineering and Remote Sensing 68: 725-734.

173. Yeh AGO, Li X (2001) Measurement and monitoring of urban sprawl in a rapidly growing region using entropy. Photogrammetric Engineering and Remote Sensing 67: 83-90.

174. Yu X, Ng C (2006) An integrated evaluation of landscape change using remote sensing and landscape metrics: a case study of Panyu, Guangzhou. International Journal of Remote Sensing 27: 1075-1092.

175. Yuan F (2008) Land-cover change and environmental impact analysis in the Greater Mankato area of Minnesota using remote sensing and GIS modelling. International Journal of Remote Sensing 29: 1169-1184.

176. Yuan F, Sawaya KE, Loeffelholz BC, Bauer ME (2005) Land cover classification and change analysis of the Twin Cities (Minnesota) Metropolitan Area by multitemporal Landsat remote sensing. Remote Sensing of Environment 98: 317-328.

177. Zeng H, Sui DZ, Li SJ (2005) Linking urban field theory with GIS and remote sensing to detect signatures of rapid urbanization on the landscape: Toward a new approach for characterizing urban sprawl. Urban Geography 26: 410-434.

178. Zhang XL, Chen J, Tan MZ, Sun YC (2007) Assessing the impact of urban sprawl on soil resources of Nanjing city using satellite images and digital soil databases. Catena 69: 16-30.

179. Zhao SQ, Da LJ, Tang ZY, Fang HJ, Song K, et al. (2006) Ecological consequences of rapid urban expansion: Shanghai, China. Frontiers in Ecology and the Environment 4: 341-346.

180. Zhao SQ, Fang JY (2004) Impact of impoldering and lake restoration on land-cover changes in Dongting Lake area, Central Yangtze. Ambio 33: 311-315.

181. Zhou WQ, Troy A, Grove M (2008) Object-based land cover classification and change analysis in the Baltimore metropolitan area using multitemporal high resolution remote sensing data. Sensors 8: 1613-1636.

**List of studies not included in our meta-analysis (with reasons noted)**

182. Abdullah SA, Nakagoshi N (2007) Forest fragmentation and its correlation to human land use change in the state of Selangor, peninsular Malaysia. Forest Ecology and Management 241: 39-48. Does not meet criterion 4.

183. Barnes CA, Roy DP (2008) Radiative forcing over the conterminous United States due to contemporary land cover land use albedo change. Geophysical Research Letters 35. Does not meet criterion 1.

184. Bergen KM, Brown DG, Rutherford JF, Gustafson EJ (2005) Change detection with heterogeneous data using ecoregional stratification, statistical summaries and a land allocation algorithm. Remote Sensing of Environment 97: 434-446. Does not meet criterion 1.

185. Chen SP, Zeng S, Xie CG (2000) Remote sensing and GIS for urban growth analysis in China. Photogrammetric Engineering and Remote Sensing 66: 593-598. Does not meet criterion 4.

186. Chen S-S, Chen L-S, Liu Q-H, Li X, Tan Q (2005) Remote sensing and GIS-based integrated analysis of coastal changes and their environmental impacts in Lingding Bay, Pearl River Estuary, South China. Ocean & Coastal Management 48: 65-83. Does not meet criterion 4.

187. Chen XL, Bao SM, Li H, Cai XB, Guo P, et al. (2007) LUCC impact on sediment loads in subtropical rainy areas. Photogrammetric Engineering and Remote Sensing 73: 319-327. Does not meet criterion 1.

188. Chen ZJ, Chen J, Shi PJ, Tamura M (2003) An IHS-based change detection approach for assessment of urban expansion impact on arable land loss in China. International Journal of Remote Sensing 24: 1353-1360. Does not meet criterion 4.

189. Coskun HG, Gulergun O, Yilmaz L (2006) Monitoring of protected bands of Terkos drinking water reservoir of metropolitan Istanbul near the Black Sea coast using satellite data. International Journal of Applied Earth Observation and Geoinformation 8: 49-60. Does not meet criterion 4.

190. Dai E, Wu SH, Shi WZ, Cheung CK, Shaker A (2005) Modeling change-pattern-value dynamics on land use: An integrated GIS and artificial neural networks approach. Environmental Assessment 36: 576-591. Does not meet criterion 1.

191. Dewidar KM (2004) Detection of land use land cover changes for the northern part of the Nile delta (Burullus region), Egypt. International Journal of Remote Sensing 25: 4079-4089. Does not meet criterion 4.

192. Du Y, Xie ZQ, Zeng Y, Shi YF, Wu JG (2007) Impact of urban expansion on regional temperature change in the Yangtze River Delta. Journal of Geographical Sciences 17: 387-398. Does not meet criterion 3.

193. Duran Z, Musaoglu N, Seker DZ (2006) Evaluating urban land use change in historical peninsula, Istanbul, by using gis and remote sensing. Fresenius Environmental Bulletin 15: 806-810. Does not meet criterion 4.

194. El-Raey M, Fouda Y, Gal P (2000) GIS for environmental assessment of the impacts of urban encroachment on Rosetta Region, Egypt. Environmental Monitoring and Assessment 60: 217-233. Does not meet criterion 4.

195. Evelyn OB, Camirand R (2003) Forest cover and deforestation in Jamaica: an analysis of forest cover estimates over time. International Forestry Review 5: 354-363. Does not meet criterion 3.

196. Gao J, Liu YS, Chen YF (2006) Land cover changes during agrarian restructuring in Northeast China. Applied Geography 26: 312-322. Does not meet criterion 1.

197. Geymen A, Baz I (2008) Monitoring urban growth and detecting land-cover changes on the Istanbul metropolitan area. Environmental Monitoring and Assessment 136: 449-459. Does not meet criterion 4.

198. Goksel C, Musaoglu N, Gurel M, Ulugtekin N, Tanik A, et al. (2006) Determination of land-use change in an urbanized district of Istanbul via remote sensing analysis. Fresenius Environmental Bulletin 15: 798-805. Does not meet criterion 4.

199. He CY, Shi PJ, Li JG, Chen J, Pan YZ, et al. (2006) Restoring urbanization process in China in the 1990s by using non-radiance calibrated DMSP/OLS nighttime light imagery and statistical data. Chinese Science Bulletin 51: 1-7. Does not meet criterion 1.

200. Helmer EH (2004) Forest conservation and land development in Puerto Rico. Landscape Ecology 19: 29-40. Does not meet criterion 3.

201. Ho SPS, Lin GCS (2004) Non-Agricultural Land Use in Post-Reform China. China Quarterly 179: 758-781. Does not meet criterion 4.

202. Huang J, Zhu L, Deng X (2007) Regional differences and determinants of built-up area expansion in China. Science in China (Series D)-Earth Sciences 50: 1835-1843. Does not meet criterion 1.

203. Jantz P, Goetz S, Jantz C (2005) Urbanization and the loss of resource lands in the Chesapeake Bay watershed. Environmental Management 36: 808-825. Does not meet criterion 1.

204. Ji CY, Liu QH, Sun DF, Wang S, Lin P, et al. (2001) Monitoring urban expansion with remote sensing in China. International Journal of Remote Sensing 22: 1441-1455. Does not meet criterion 1.

205. Jones KB, Neale AC, Wade TG, Wickham JD, Cross CL, et al. (2001) The consequences of landscape change on ecological resources: An assessment of the United States mid-Atlantic region, 1973-1993. Ecosystem Health 7: 229-242. Does not meet criterion 1.

206. Kavzoglu T (2008) Determination of environmental degradation due to urbanization and industrialization in Gebze, Turkey. Environmental Engineering Science 25: 429-438. Does not meet criterion 4.

207. Kaya S (2007) Multitemporal analysis of rapid urban growth in Istanbul using remotely sensed data. Environmental Engineering Science 24: 228-233. Does not meet criterion 4.

208. Kaya S, Curran PJ (2006) Monitoring urban growth on the European side of the Istanbul metropolitan area: A case study. International Journal of Applied Earth Observation and Geoinformation 8: 18-25. Does not meet criterion 4.

209. Kolar J (2001) Land cover accounting. International Journal of Environment and Pollution 15: 695-713. Does not meet criterion 3.

210. Kucukmehmetoglu M, Geymen A (2008) Measuring the spatial impacts of urbanization on the surface water resource basins in Istanbul via remote sensing. Environmental Monitoring and Assessment 142: 153-169. Does not meet criterion 4.

211. Lenney MP, Woodcock CE, Collins JB, Hamdi H (1996) The status of agricultural lands in Egypt: The use of multitemporal NDVI features derived from Landsat TM. Remote Sensing of Environment 56: 8-20. Does not meet criterion 4.

212. Li X, Yeh AGO (2004) Analyzing spatial restructuring of land use patterns in a fast growing region using remote sensing and GIS. Landscape and Urban Planning 69: 335-354. Does not meet criterion 3.

213. Li Y, Zhao SQ, Zhao K, Xie P, Fang JY (2006) Land-cover changes in an urban lake watershed in a mega-city, Central China. Environmental Monitoring and Assessment 115: 349-359. Does not meet criterion 4.

214. Li YC (2008) Land cover dynamic changes in northern China: 1989-2003. Journal of Geographical Sciences 18: 85-94. Does not meet criterion 1.

215. Liu H, Zhou Q (2004) Accuracy analysis of remote sensing change detection by rule-based rationality evaluation with post-classification comparison. International Journal of Remote Sensing 25: 1037-1050. Does not meet criterion 4.

216. Liu JY, Tian HQ, Liu ML, Zhuang DF, Melillo JM, et al. (2005) China's changing landscape during the 1990s: Large-scale land transformations estimated with satellite data. Geophysical Research Letters 32: L02405 Artn l02405. Does not meet criterion 1.

217. Liu JY, Zhan JY, Deng XZ (2005) Spatio-temporal patterns and driving forces of urban land expansion in china during the economic reform era. Ambio 34: 450-455. Does not meet criterion 1.

218. Liu YS, Wang DW, Gao J, Deng W (2005) Land use/cover changes, the environment and water resources in Northeast China. Environmental Management 36: 691-701. Does not meet criterion 1.

219. Lo CP, Quattrochi DA (2003) Land-use and land-cover change, urban heat island phenomenon, and health implications: A remote sensing approach. Photogrammetric Engineering and Remote Sensing 69: 1053-1063. Does not meet criterion 2.

220. Lo CP, Yang XJ (2002) Drivers of land-use/land-cover changes and dynamic modeling for the Atlanta, Georgia Metropolitan Area. Photogrammetric Engineering and Remote Sensing 68: 1073-1082. Does not meet criterion 2.

221. Lopez TD, Aide TM, Thomlinson JR (2001) Urban expansion and the loss of prime agricultural lands in Puerto Rico. Ambio 30: 49-54. Does not meet criterion 3.

222. Maktav D, Erbek FS (2005) Analysis of urban growth using multi-temporal satellite data in Istanbul, Turkey. International Journal of Remote Sensing 26: 797-810. Does not meet criterion 4.

223. Mena CF (2008) Trajectories of land-use and land-cover in the northern Ecuadorian Amazon: Temporal composition, spatial configuration, and probability of change. Photogrammetric Engineering and Remote Sensing 74: 737-751. Does not meet criterion 4.

224. Milesi C, Elvidge CD, Nemani RR, Running SW (2003) Assessing the impact of urban land development on net primary productivity in the southeastern United States. Remote Sensing of Environment 86: 401-410. Does not meet criterion 1.

225. Mundia CN, Aniya M (2005) Analysis of land use/cover changes and urban expansion of Nairobi city using remote sensing and GIS. International Journal of Remote Sensing 26: 2831-2849. Does not meet criterion 3.

226. Musaoglu N, Coskun M, Kocabas V (2005) Land use change analysis of Beykoz-Istanbul by means of satellite images and GIS. Water Science and Technology 51: 245-251. Does not meet criterion 4.

227. Musaoglu N, Gurel M, Ulugtekin N, Tanik A, Seker DZ (2006) Use of remotely sensed data for analysis of land-use change in a highly urbanized district of mega city, Istanbul. Journal of Environmental Science and Health Part a-Toxic/Hazardous Substances & Environmental Engineering 41: 2057-2069. Does not meet criterion 4.

228. Ouyang TP, Kuang YQ, Hu ZY, Sun B (2005) Urbanization in the Pearl River Delta Economic Zone, China. International Journal of Sustainable Development and World Ecology 12: 48-54. Does not meet criterion 3.

229. Qi SZ, Luo F (2006) Land-use change and its environmental impact in the Heihe River Basin, arid northwestern China. Environmental Geology 50: 535-540. Does not meet criterion 1.

230. Qi Y, Henderson M, Xu M, Chen J, Shi PJ, et al. (2004) Evolving core-periphery interactions in a rapidly expanding urban landscape: The case of Beijing. Landscape Ecology 19: 375-388. Does not meet criterion 4.

231. Qian LX, Cui HS, Jie C (2006) Impacts of land use and cover change on land surface temperature in the Zhujiang Delta. Pedosphere 16: 681-689. Does not meet criterion 4.

232. Quan B, Zhu H-J, Chen S-L, Romkens MJM, Li B-C (2007) Land Suitability Assessment and Land Use Change in Fujian Province, China. Pedosphere 17: 493-504. Does not meet criterion 1.

233. Ramadan E, Feng X-z, Cheng Z (2004) Satellite remote sensing for urban growth assessment in Shaoxing City, Zhejiang Province. Journal of Zhejiang University Science 5: 1095-1101. Does not meet criterion 4.

234. Robinson L, Newell JP, Marzluff JA (2005) Twenty-five years of sprawl in the Seattle region: growth management responses and implications for conservation. Landscape and Urban Planning 71: 51-72. Does not meet criterion 4.

235. Salas WA, Boles SH, Frolking S, Xiao X, Li C (2003) The perimeter/area ratio as an index of misregistration bias in land cover change estimates. International Journal of Remote Sensing 24: 1165-1170. Does not meet criterion 3.

236. Salem BB, Elcibahy A, Elraey M (1995) Detection of land-cover classes in agroecosystems of northern Egypt by remote-sensing. International Journal of Remote Sensing 16: 2581-2594. Does not meet criterion 4.

237. Seto KC, Fragkias M (2005) Quantifying spatiotemporal patterns of urban land-use change in four cities of China with time series landscape metrics. Landscape Ecology 20: 871-888. Does not meet criterion 3.

238. Seto KC, Woodcock CE, Song C, Huang X, Lu J, et al. (2002) Monitoring land-use change in the Pearl River Delta using Landsat TM. International Journal of Remote Sensing 23: 1985-2004. Does not meet criterion 3.

239. Shi PJ, Yuan Y, Zheng J, Wang JA, Ge Y, et al. (2007) The effect of land use/cover change on surface runoff in Shenzhen region, China. CATENA 69: 31-35. Does not meet criterion 4.

240. Stewart DJ, Yin ZY, Bullard SM, MacLachlan JT (2004) Assessing the spatial structure of urban and population growth in the Greater Cairo area, Egypt: A GIS and imagery analysis approach. Urban Studies 41: 95-116. Does not meet criterion 4.

241. Streets D, Chung C, Krummel J, Su H (1995) Remote sensing of global change: Growth in China's Jiangsu province. International Journal of Sustainable Development and World Ecology 2: 257-266. Does not meet criterion 3.

242. Sultan M, Fiske M, Stein T, Gamal M, Hady YA, et al. (1999) Monitoring the urbanization of the Nile Delta, Egypt. Ambio 28: 628-631. Does not meet criterion 4.

243. Tan MH, Li XB, Lu CH (2005) Urban land expansion and arable land loss of the major cities in China in the 1990s. Science in China (Series D)-Earth Sciences 48: 1492-1500. Does not meet criterion 3.

244. Tan MH, Li XB, Xie H, Lu CH (2005) Urban land expansion and arable land loss in China - a case study of Beijing-Tianjin-Hebei region. Land Use Policy 22: 187-196. Does not meet criterion 3.

245. Tang J, Wang L, Yao Z (2007) Spatio-temporal urban landscape change analysis using the Markov chain model and a modified genetic algorithm. International Journal of Remote Sensing 28: 3255-3271. Does not meet criterion 4.

246. Tang JM, Wang L, Yao ZJ (2006) Analyzing urban sprawl spatial fragmentation using multi-temporal satellite images. GIScience & Remote Sensing 43: 218-232. Does not meet criterion 4.

247. Tang JM, Wang L, Yao ZJ (2008) Analyses of urban landscape dynamics using multi-temporal satellite images: A comparison of two petroleum-oriented cities. Landscape and Urban Planning 87: 269-278. Does not meet criterion 4.

248. Wang SQ, Tian HQ, Liu JY, Zhuang DF, Zhang SW, et al. (2002) Characterization of changes in land cover and carbon storage in Northeastern China: An analysis based on Landsat TM data. Science in China (Series C)-Life Sciences 45: 40-+. Does not meet criterion 1.

249. Wang YQ, Tobey J, Bonynge G, Nugranad J, Makota V, et al. (2005) Involving geospatial information in the analysis of land-cover change along the Tanzania coast. Coastal Management 33: 87-99. Does not meet criterion 1.

250. Wang ZM, Zhang B, Zhang SQ, Li XY, Liu DW, et al. (2006) Changes of land use and of ecosystem service values in Sanjiang Plain, northeast China. Environmental Monitoring and Assessment 112: 69-91. Does not meet criterion 1.

251. Weng Q, Yang S (2004) Managing the adverse thermal effects of urban development in a densely populated Chinese city. Journal of Environmental Management 70: 145-156. Does not meet criterion 4.

252. Weng QH (2002) Land use change analysis in the Zhujiang Delta of China using satellite remote sensing, GIS and stochastic modelling. Journal of Environmental Management 64: 273-284. Does not meet criterion 2.

253. Weng QH, Yang SH (2006) Urban air pollution patterns, land use, and thermal landscape: An examination of the linkage using GIS. Environmental Monitoring and Assessment 117: 463-489. Does not meet criterion 2.

254. Woomer PL, Tieszen LL, Tappan G, Toure A, Sall M (2004) Land use change and terrestrial carbon stocks in Senegal. Journal of Arid Environments 59: 625-642. Does not meet criterion 1.

255. Xian G, Crane M (2005) Assessments of urban growth in the Tampa Bay watershed using remote sensing data. Remote Sensing of Environment 97: 203-215. Does not meet criterion 2.

256. Xian G, Crane M (2006) An analysis of urban thermal characteristics and associated land cover in Tampa Bay and Las Vegas using Landsat satellite data. Remote Sensing of Environment 104: 147-156. Does not meet criterion 2.

257. Xu C, Liu MS, Zhang C, An SQ, Yu W, et al. (2007) The spatiotemporal dynamics of rapid urban growth in the Nanjing metropolitan region of China. Landscape Ecology 22: 925-937. Does not meet criterion 4.

258. Yener H, Koc A (2006) Monitoring changes in forest and other land use forms in Istanbul. Journal of Environmental Biology 27: 77-83. Does not meet criterion 4.

259. Yildirim H, Ozel ME, Divan NJ (2002) Satellite monitoring of land cover/land use change over 15 years and its impact on the environment in Gebze/Kocaeli - Turkey. Turkish Journal of Agriculture and Forestry 26: 161-170. Does not meet criterion 4.

260. Yu XJ, Ng CN (2007) Spatial and temporal dynamics of urban sprawl along two urban-rural transects: A case study of Guangzhou, China. Landscape and Urban Planning 79: 96-109. Does not meet criterion 3.

261. Zhang H, Ma WC, Wang XR (2008) Rapid urbanization and implications for flood risk management in hinterland of the Pearl River Delta, China: The Foshan study. Sensors 8: 2223-2239. Does not meet criterion 2.

262. Zhang H, Wang XR (2007) Land-use dynamics and flood risk in the hinterland of the Pearl River Delta: The case of Foshan City. International Journal of Sustainable Development and World Ecology 14: 485-492. Does not meet criterion 3.

263. Zhang Q, Wang J, Peng X, Gong P, Shi P (2002) Urban built-up land change detection with road density and spectral information from multi-temporal Landsat TM data. International Journal of Remote Sensing 23: 3057-3078. Does not meet criterion 4.

264. Zhao B, Kreuter U, Li B, Ma ZJ, Chen JK, et al. (2004) An ecosystem service value assessment of land-use change on Chongming Island, China. Land Use Policy 21: 139-148. Does not meet criterion 4.

265. Zhao B, Nakagoshi N, Chen JK, Kong LY (2003) The impact of urban planning on land use and land cover in Pudong of Shanghai, China. Journal of Environmental Sciences-China 15: 205-214. Does not meet criterion 4.
