# Supplementary material for: A Meta-Analysis of Global Urban Land Expansion
Source: PLoS One. 2011 Aug 18;6(8):e23777. doi: 10.1371/journal.pone.0023777 (PMC3158103; doi:10.1371/journal.pone.0023777)
Supplement: Text S3 — Meta-analysis methodology. (DOCX) [file pone.0023777.s008.docx]

**A Meta-Analysis of Global Urban Land Expansion**

**Text S3. Meta-analysis methodology**

Our meta-analytic method for integrating rates and magnitudes of urban land-use change quantitatively combines and summarizes research results across individual and independent studies (Hedges and Olkins, 1985). The results of individual studies are converted into a standard metric, which is called “effect size”. Commonly used measures of effect size are standardized difference between means of experimental and control groups and the Pearson product moment correlation coefficient - other metrics may also be more appropriate depending on the nature of the study (Osenberg et al., 1999). Confidence intervals generated by bootstrapping techniques are used to bound the common estimate.

The effect size of interest in this meta-analysis is the annual rate of urban land-use change. Our metric is the geometric rate of change AGR = 100*(1+(UE_end_/UE_start_)^(1/d)^) where *UE_start_* is the extent of the urban area at the initial time period, *UE_end_* the extent of the study at the final time period and d: the time span of the study in years. Thus in our calculations of this effect size, we account for differences in the length of time intervals in between the monitoring of urban areas. As a simplifying assumption and to be able to include those papers that simply report the year an image is taken, we used not the exact date but the first day of the year an image is taken in our calculations. Following our aggregation method for world regions, we respectively calculate the average annual rate of change for world regions in decadal time intervals (e.g., for North America during the decades 1970-1979, 1980-1989, 1990-1999). We also generated confidence intervals through the technique of bootstrapping.

The size of study areas, when not reported explicitly in the text, is gathered from tables or figures (i.e., maps) when possible. For observations derived from the Angel et al. (2005) report (which does not produce the total area considered for each city) we define the urban extent of the final year of the study as the estimate of the entire study area (being the lowest possible estimate of the study area). This does not affect our major findings and is done only for use in calculating how much of the Earth's ice-free surface has been analyzed in the peer-reviewed literature. When there are more than one study on the same location the averages for the magnitudes and rates of urban expansion are derived from the values reported in these studies.

In decadal estimates, the starting year of each decade is used to estimate total urban land and change in urban land that occurred in each decade. The decadal rates are estimated in a similar fashion.

In decadal estimates, urban land at the start of each decade is calculated as follows: It is assumed that for each study site, the average annual rate of change found for the analysis period of that study holds for the rest of our study’s analysis window (i.e. 1970-1999). For this, the average annual rate of change extracted from the studies is extrapolated forward and/or backward in time. In case it is possible to extract more than one rate estimate for different portions of the study period, the rate estimates closest to the extrapolated decade is used.

For any study, if it is not possible to estimate average annual rate of change at least for 4 years for a decadal interval, no average annual rate of change estimate is assigned to that interval. This assumption leads to the “decadal” interval 2000-present having very small numbers –or none– of average annual rate estimates for any region. This sets our analysis window to the span of 1970-1999.

There is no weighting involved in any of the estimates except that the interval of each annual rate of change estimate is used as weights in coming up with aggregate and decadal average annual rate of change estimates. We considered weighting with urban extents; however, as any such weighting would bias the estimates towards larger urban areas we decided against it.

Furthermore, we used the statistical technique of nonparametric bootstrapping to estimate a measure of uncertainty over the rates of change by region. Bootstrapping is a computer-intensive practice of estimating properties of an estimator (such as its variance) by measuring those properties when sampling from an approximating distribution (Chernick, 1999). In a particular bootstrap sample, a given observation from the original dataset may appear once, more than once, or not at all. This, in effect, simulates what would happen if a new experiment were conducted. For the full 30 year period regional datasets and the decadal period regional datasets, we resample the data with replacement keeping the size of the resample equal to the size of each of the original data sets. Then we compute the mean value from the resample for each region and time period. We repeat this resampling technique 1000 times to derive an estimate of the bootstrap distribution of the means of each region and time period. We report the quartiles of the bootstrap distribution of the means of each region and time period.

We used a basic multivariate regression within a meta-analytic framework through multi-level regression on a pooled dataset. Our dependent variable is a single annualized rate for each time period in each study. This is statistically better than treating each year as in independent observation but observations within a paper or place would still likely exhibit correlation due to their serial nature and study particularities.
